# Global screening for Critical Habitat in the terrestrial realm

**DOI:** 10.1371/journal.pone.0193102

**Published:** 2018-03-22

**Authors:** Kerstin M. Brauneder, Chloe Montes, Simon Blyth, Leon Bennun, Stuart H. M. Butchart, Michael Hoffmann, Neil D. Burgess, Annabelle Cuttelod, Matt I. Jones, Val Kapos, John Pilgrim, Melissa J. Tolley, Emma C. Underwood, Lauren V. Weatherdon, Sharon E. Brooks

**Affiliations:** 1 UN Environment World Conservation Monitoring Centre (UNEP-WCMC), Cambridge, United Kingdom; 2 The Biodiversity Consultancy, Cambridge, United Kingdom; 3 Department of Zoology, University of Cambridge, Cambridge, United Kingdom; 4 BirdLife International, Cambridge, United Kingdom; 5 Conservation Programmes, Zoological Society of London, London, United Kingdom; 6 IUCN Global Species Programme, International Union for the Conservation of Nature, Cambridge, United Kingdom; 7 Department of Environmental Science and Policy, University of California, Davis, California, United States of America; Kerala Forest Research Institute, INDIA

## Abstract

Critical Habitat has become an increasingly important concept used by the finance sector and businesses to identify areas of high biodiversity value. The International Finance Corporation (IFC) defines Critical Habitat in their highly influential Performance Standard 6 (PS6), requiring projects in Critical Habitat to achieve a net gain of biodiversity. Here we present a global screening layer of Critical Habitat in the terrestrial realm, derived from global spatial datasets covering the distributions of 12 biodiversity features aligned with guidance provided by the IFC. Each biodiversity feature is categorised as ‘likely’ or ‘potential’ Critical Habitat based on: 1. Alignment between the biodiversity feature and the IFC Critical Habitat definition; and 2. Suitability of the spatial resolution for indicating a feature’s presence on the ground. Following the initial screening process, Critical Habitat must then be assessed in-situ by a qualified assessor. This analysis indicates that a total of 10% and 5% of the global terrestrial environment can be considered as likely and potential Critical Habitat, respectively, while the remaining 85% did not overlap with any of the biodiversity features assessed and was classified as ‘unknown’. Likely Critical Habitat was determined principally by the occurrence of Key Biodiversity Areas and Protected Areas. Potential Critical Habitat was predominantly characterised by data representing highly threatened and unique ecosystems such as ever-wet tropical forests and tropical dry forests. The areas we identified as likely or potential Critical Habitat are based on the best available global-scale data for the terrestrial realm that is aligned with IFC’s Critical Habitat definition. Our results can help businesses screen potential development sites at the early project stage based on a range of biodiversity features. However, the study also demonstrates several important data gaps and highlights the need to incorporate new and improved global spatial datasets as they become available.

## Introduction

Improved management of business and supply chain impacts is increasingly recognised as a global priority to avert environmental crises and achieve a sustainable global economy [1]. Accordingly, governments, conservation organisations, shareholders and consumers have been exerting pressure on the business community to identify and manage their impacts on biodiversity [2]. Simultaneously, within the business community, there is a growing understanding of how biodiversity and the services it provides can present both risks and opportunities for business and society more broadly [3].

Businesses across multiple sectors and scales have adopted a wide range of strategies to identify and mitigate biodiversity impacts [4,5]. Business operations are guided by a company's own standards, national legislation, and criteria set by voluntary standards systems and finance institutions. One of the most influential financial institutions, particularly within large-scale infrastructure and extractive sectors, is the International Finance Corporation (IFC). In 2012, the IFC launched its current Performance Standard 6 (IFC PS6), relating to Biodiversity Conservation and Sustainable Management of Living Natural Resources [6]. IFC PS6 was developed through extensive consultation with leading biodiversity conservation groups and experts and is frequently considered a blueprint of best practices related to biodiversity for business. For instance, Decision XI/7 at the 11th Conference of the Parties of the Convention on Biological Diversity “call[ed] upon businesses to consider the revised 2012 International Finance Corporation Performance Standards” [7]. PS6 not only defines the requirements for biodiversity performance of companies financed by the IFC, but also acts as a standard for projects undertaken by other global finance groups such as the 90 Equator Principle Finance Institutions (EPFIs) [8]. IFC’s influence is global, with nearly US$19 billion invested across the world in the 2016 Financial Year, while the EPFIs cover over 70 percent of international Project Finance debt in emerging markets [9].

Three key objectives are defined under PS6: to protect and conserve biodiversity; to maintain the benefits from ecosystem services; and to promote the sustainable management of living natural resources through the adoption of practices that integrate conservation needs and development priorities [10]. To achieve these objectives, PS6 requires the identification of risks and impacts arising from projects based on the types of habitat in which they occur. Critical Habitat represents areas of high biodiversity value based on five criteria that address habitat of significant importance to threatened, endemic, congregatory and migratory species, threatened or unique ecosystems, and key evolutionary processes. Furthermore, PS6 requires projects to achieve net gains in the biodiversity values for which the Critical Habitat was designated.

The presence or close proximity of Critical Habitat to business operations can have substantial implications for companies. It can influence access to finance at different stages of the project lifecycle, and the degree of mitigation effort required. Companies therefore have a keen interest in assessing the likelihood of Critical Habitat being present within or near project sites. Having an early indication of the possible presence of Critical Habitat can help businesses focus their efforts in subsequent on-site assessments with qualified personnel (for general guidance see [11]) and can support mitigation planning early in the project lifecycle.

Over recent years, several tools have been developed to aid the assessment and screening of the biodiversity value of sites, including the Integrated Biodiversity Assessment Tool [12] and the Local Ecological Footprint Tool [13]. Additionally, analytical spatial tools have been developed to support conservation planning (e.g. Marxan, [14]; and MarineMap, [15]) and to assess the value of ecosystem services (e.g. InVEST, [16]; and ARIES [17]). These tools can be useful for estimating biodiversity and ecosystem service values which can inform impact assessments and associated management plans. However, none of these tools are designed to help identify Critical Habitat as stipulated by IFC PS6. In 2015, Martin et al. [18] produced the first screening layer of Critical Habitat for the marine environment but, until now, there has been no corresponding analysis for the terrestrial realm. This study addresses this gap by developing a global screening layer derived from the spatial overlay of terrestrial biodiversity data that align with one or more of the PS6 criteria defining Critical Habitat.

The application of this work is largely aimed at the global finance sector, principally the IFC, the 90 EPFIs, and large-scale business operations that are seeking project finance. It is likely to be of particular interest to the extractives sector, where a number of companies have started to adopt elements of IFC Performance Standard 6 as part of their internal safeguards. We also foresee a wider application across both private and public finance as other multilateral finance institutions increasingly align their definition and thresholds of Critical Habitat with IFC PS6, for example the World Bank’s revised Environment and Social Framework [19]. This work therefore supports efforts to make spatially explicit biodiversity data available and relevant for improved decisions on development.

## Methodology

### Critical Habitat criteria and scenarios

IFC PS6 defines Critical Habitat as areas with high biodiversity value, based on a set of five criteria (Table 1). Critical Habitat may also be triggered by other recognized high biodiversity values, described in detail in the IFC Guidance Note 6 (GN6) [10]. These are referred to here as scenarios A and B following the approach in Martin et al. [18]. Scenario A includes additional biodiversity values that are not included within the criteria, for example “Ecosystems of known special significance to Endangered or Critically Endangered species for climate adaptation purpose”. Scenario B refers to internationally or nationally recognized areas of high biodiversity value that are explicitly mentioned within GN6, such as UNESCO natural World Heritage sites and Wetlands of International Importance under the Ramsar Convention (for more detail see S1 Table in [18]). Within GN6, Criteria 1–3 are further divided into Tier 1 and Tier 2 Critical Habitat depending on the relative vulnerability and irreplaceability of the biodiversity present. Mapping these tiers would require more refined datasets than those that are available globally and, consequently, they are not indicated in the screening layer developed here.

**Table 1:** Critical Habitat criteria and scenarios.

Criteria and scenarios	Description of biodiversity values
Criterion 1	Habitat of significant importance to Critically Endangered and/or Endangered species
Criterion 2	Habitat of significant importance to endemic and/or restricted-range species
Criterion 3	Habitat supporting globally significant concentrations of migratory species and/or congregatory species
Criterion 4	Highly threatened and/or unique ecosystems
Criterion 5	Areas associated with key evolutionary processes
Scenario A	Other recognized high biodiversity values that might also support a Critical Habitat designation
Scenario B	Internationally and/or nationally recognized areas of high biodiversity value that in general will likely qualify as Critical Habitat

Biodiversity values recognized under the Critical Habitat designation are categorized under five criteria within the International Finance Corporation’s Performance Standard 6 and its associated Guidance Note 6. Scenarios A and B are also based on the IFC standard and guidance note and grouped following S1 Table in Martin et al. [18].

### Data screening and classification

Relevant spatial datasets were identified through consultation among the authors and other experts based on the following criteria adapted from Martin et al. [18]: (i) direct relevance to one or more Critical Habitat criteria/scenarios; (ii) global in extent; (iii) assembled using a standardised protocol; (iv) the best available data for the biodiversity feature of interest; and (v) sufficiently high resolution to indicate presence of biodiversity on the ground at scales of relevance to business operations. Where data were not published or available for use at the time of the analysis, they were excluded from the study (S2 Table).

**Fig 1:**
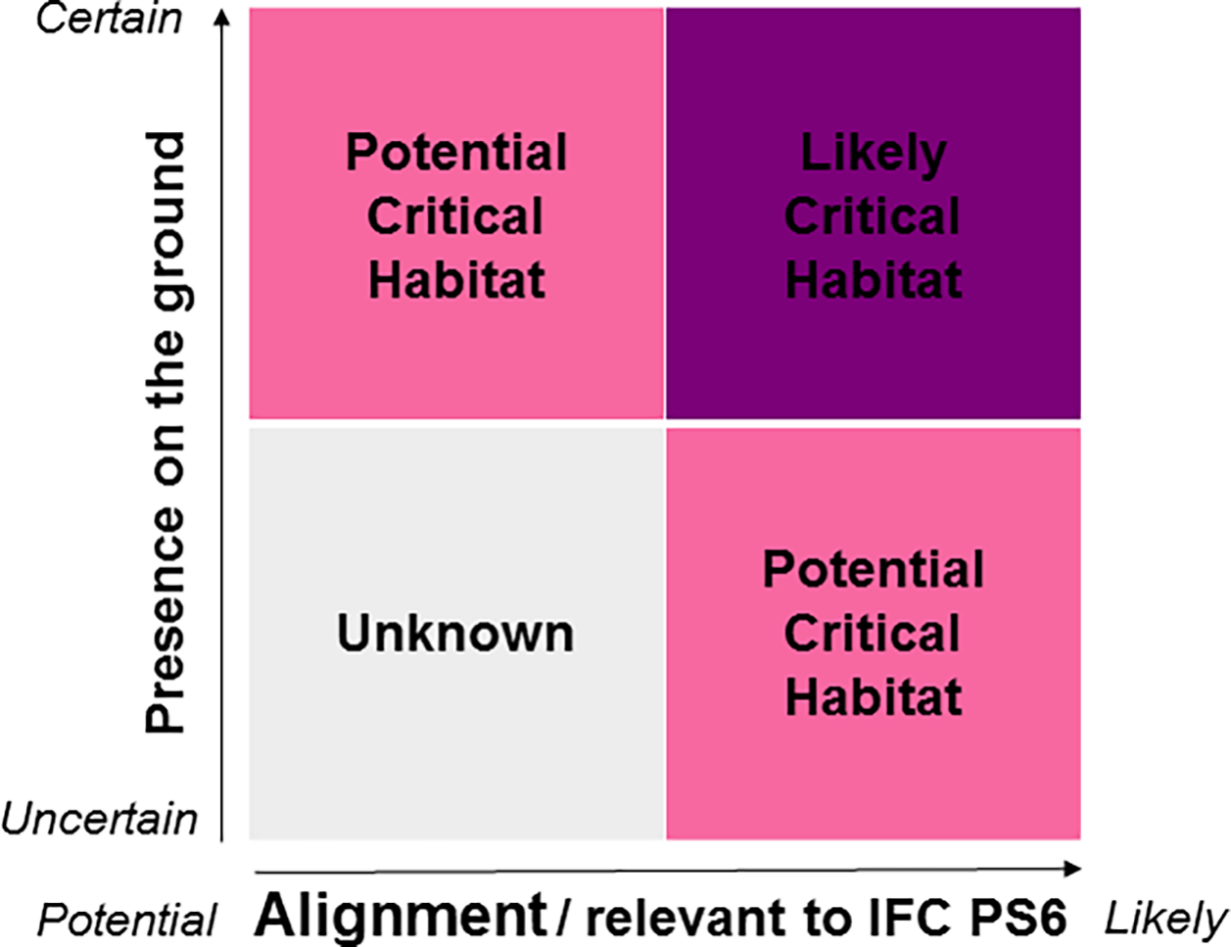
Screening layer classification scheme. Classification of data subsets as likely or potential Critical Habitat is based on the strength of alignment with IFC PS6 criteria and scenarios and the spatial resolution of the data (adapted from Martin et al. [18]).

Datasets retained for the analysis were classified as ‘likely’ or ‘potential’ Critical Habitat based on two variables: alignment of the dataset with the Critical Habitat definition, and spatial resolution of the dataset indicating presence on the ground (Fig 1). Biodiversity features represented by data with strong alignment with one or more Critical Habitat criteria or scenarios A or B, and high spatial resolution were classified as likely Critical Habitat. Where alignment with Critical Habitat criteria and scenarios was less strong or the spatial resolution of the dataset was coarser, features were mapped as potential Critical Habitat. These categories of Critical Habitat indicate the likelihood of finding Critical Habitat in subsequent on-site assessments. Areas outside of likely or potential Critical Habitat are classified as *‘*Unknown’. These areas include both Critical Habitat for which no suitable global-scale biodiversity data currently exist and areas that do not qualify as Critical Habitat based on their biodiversity values.

### Data processing and spatial analysis

The identification of terrestrial Critical Habitat follows the methodology developed for screening Critical Habitat in the marine realm outlined by Martin et al. [18]. The analysis uses the same likely and potential Critical Habitat classification scheme, resolution, and shoreline delineation (GSHHG version 2.2.2, [20]). The screening exercise retained datasets in both vector and raster format, all of which were converted into raster layers of 1 km grid cell size in cylindrical equal-area projection. Vector data represent the location and extent of features using points, lines and polygons. Raster data consists of a matrix of cells, where each pixel contains a value representing information. During the vector to raster conversion, likely or potential grid-cell values were assigned upon intersection with the relevant point, polygon or line features, irrespective of the area of overlap or frequency of features. The 1 km grid cell size ensures that point features are visible on the final layer, and that boundaries of point and polygon features are preserved. The final map is a composite of all underlying layers produced by successively combining individual rasters and retaining the highest class for overlapping grid cells. In hierarchical order: likely grid cells (purple in Fig 1) were retained over potential grid cells (pink), in turn being retained over unknown grid cells (grey). A key benefit of using the same methodology for both terrestrial and marine realms is that it can facilitate the combination of terrestrial and marine Critical Habitat screening layers in the future.

## Results

### Selected datasets

Thirty-six data sources were identified as suitable to review for Critical Habitat. Of these, 12 datasets (Table 2) relating to 12 biodiversity features (Table 3) were retained for use in the screening layer. These include datasets that represent biodiversity features explicitly referred to in GN6, those directly aligned with the PS6 criteria, and those for which the existing scientific literature provides an evidence-based justification for alignment with the PS6 criteria (S1 Table). Note that a single dataset may provide spatial information on multiple biodiversity features, which may be classified differently as likely or potential depending on their alignment with IFC PS6 criteria and scenarios.

**Table 2:** Datasets incorporated into the Critical Habitat screening layer.

Dataset	Year / version	Update frequency	Native format	Biodiversity feature(s)	Ref.
Ever wet tropical forests	2015	N/A	Raster	Ever wet tropical forests	[30]
World Database of Key Biodiversity Areas	Dec 2016	Annual	Point (8%) Polygon (92%)	Alliance for Zero Extinction sites (AZEs), Important Bird and Biodiversity Areas IBAs), Key Biodiversity Areas (KBAs)	[24]
Global Distribution of Mangroves	2015 (v1.3)	Intermittent	Polygon	Mangroves	[28]
Global Distribution of Saltmarsh	2017 (v4)	Intermittent	Point (0.2%) Polygon (99.8%)	Saltmarshes	[29]
Global Distribution of Sea Turtle Nesting Sites	1999	N/A	Line	Sea turtle nesting sites	[35]
Global distribution of Tropical dry forest	2006	N/A	Polygon	Tropical dry forest	[31]
Irreplaceable Protected Areas	2013	N/A	Polygon	Irreplaceable Protected Areas	[21]
IUCN Red List of Threatened Species	2016–2	Annual	Polygon	Threatened species	[23]
Tiger Conservation Landscapes	2010	N/A	Polygon	Tiger Conservation Landscapes	[36]
Cloud forests	2004	N/A	Polygon	Tropical Montane Cloud Forest	[32]
Global Directory of Tropical Montane Cloud Forests	1997	N/A	Point	Tropical Montane Cloud Forest	[33]
World Database on Protected Areas	Feb 2017	Monthly	Point (5%) Polygon (95%)	National-level Protected Areas; World Heritage sites; Ramsar sites	[22]

‘intermittent’ update frequency indicates less than annual updates, while ‘N/A’ identifies no formal, known update strategy for the dataset. “Native format” refers to the original format of the data and the proportion of features with polygon versus point data

**Table 3:** Biodiversity features included in the analysis, their alignment with IFC PS6 Critical Habitat criteria and scenarios, and classification as ‘likely’ or ‘potential’ Critical Habitat.

Biodiversity features	Designation criterion / Trigger	IFC PS6 criteria / scenario							Classi-fication
		1	2	3	4	5	A	B	
Key Biodiversity Areas (KBAs)	Vulnerability criterion for CR species	L							Likely
	Vulnerability criterion for EN species	L							Likely
	Irreplaceability criterion, sub-criterion a		L						Likely
	Irreplaceability criterion, sub-criteria b, c and d			L					Likely
	Irreplaceability criterion, sub-criterion e				P				Potential
Alliance for Zero Extinction sites (AZEs)		L	L	L					Likely
Important Bird and Biodiversity Areas (IBAs)	Criterion A1 for CR species	L							Likely
	Criterion A1 for EN species	L							Likely
	Criterion A2		P						Potential
	Criterion A4			L					Likely
	Criterion A3				P				Potential
	CR and EN species which occupy 10 or fewer sites	L							Likely
Protected areas	IUCN management categories Ia, Ib, II							L	Likely
	Natural and mixed World Heritage sites							L	Likely
	Irreplaceable protected areas							L	Likely
	Ramsar sites designated under criteria 1, 3				L				Likely
	Ramsar sites designated under criterion 2	L							Likely
	Ramsar sites designated under criteria 5, 6			L					Likely
	Ramsar sites designated under criteria 4, 7, 8, 9			P					Potential
	All Ramsar sites							L	Likely
	Protected Areas overlapping with ≥10% of the global range of a CR or EN species	P							Potential
	Protected Areas overlapping with ≥95% of the global range of endemic or restricted-range species (range ≤ 50,000 km^2^)		P						Potential
Tiger Conservation Landscapes	Source sites	L							Likely
	Potential source sites	P							Potential
Distributions of Threatened species	CR species qualifying under IUCN Red List Criterion D	L							Likely
	EN species qualifying under IUCN Red List Criterion D	P							Likely
	VU species qualifying under IUCN Red List criterion D2		P						Likely
Sea turtle nesting sites	CR species	L							Likely
	EN species	L							Likely
	All species			P	P				Potential
Mangroves					L				Likely
Saltmarshes					L				Likely
Ever wet tropical forests					P				Potential
Tropical dry forests					P				Potential
Tropical montane cloud forests					P				Potential

”L”: Likely Critical Habitat; “P”: Potential Critical Habitat; “CR”: Critically Endangered; “EN”: Endangered; “VU”: Vulnerable

The datasets can be broadly categorised as: 1) protected areas, 2) Key Biodiversity Areas, 3) threatened ecosystems, 4) critical sites for selected species (tigers and sea turtles), and 5) the distributions of threatened species qualifying under IUCN Red List criterion D. Detailed justifications for the exclusion or retention of individual datasets and features are provided in the supplementary materials (S1 Table and S2 Table respectively). Below we describe these five types of datasets and how, for some datasets, we extracted subsets of the data where there is alignment with Critical Habitat criteria and scenarios.

1. Protected areas. The specific subsets of protected areas included are: natural and mixed World Heritage Sites, Wetlands of International Importance (Ramsar sites), and protected areas under IUCN management categories Ia, Ib and II. These are explicitly referred to in GN6, and always treated as Critical Habitat (paragraph GN115 in [10]). GN6 indicates that areas under IUCN management categories III-VI and areas for which the IUCN management category was not reported or not assigned may qualify as Critical Habitat if underlying biodiversity values align with Critical Habitat criteria. In this study, the top 100 irreplaceable protected areas worldwide as identified by Le Saout et al. [21] were also included, which reflect GN6’s ‘*Areas determined to be irreplaceable*’ (GN57). Additionally, a subset of all protected areas in the World Database on Protected Areas (WDPA) [22] was included based on an inferred alignment with PS6 criteria 1 and 2. The subset comprises protected areas that overlap with ≥10% of the range of one or more Critically Endangered (CR) or Endangered (EN) species within the IUCN Red List ([23]), or ≥95% of the range of one or more restricted-range species. Restricted range species were defined as those with a range of less than 50,000 km^2^ (IFC GN6), and the percent range overlap was used as a proxy for percent population. It is recognised that percent range is an imperfect measure of percent population due to uneven distributions of individuals, but this was considered to be the best available approach to indicate alignment of protected areas for which the underlying basis for designation is not reported.
2. Key Biodiversity Areas, including Important Bird and Biodiversity Areas (IBAs) and Alliance for Zero Extinction sites (AZEs). KBAs are identified using threshold-based criteria [24], and subsets of the KBA dataset were extracted where these aligned with Critical Habitat criteria. A global standard for the identification of KBAs was recently published [25], but we used the criteria and thresholds for identifying KBAs specified in Langhammer et al. [26] as all non-IBA and non-AZE KBAs identified to date have used these criteria. In addition, they were taken into consideration during the development of the PS6 criteria and consequently display direct alignment. This is expected to change in view of the new KBA Standard and a proposed alignment between the new KBA criteria and PS6 is provided in S3 Table.
3. Threatened ecosystem datasets. GN6 [10] does not provide detailed numeric thresholds to identify highly threatened and unique ecosystems, but points to the IUCN Red List of Ecosystems (RLE) as a suitable standard in development (paragraph GN62). The RLE provides quantitative guidelines to assess the threatened status of ecosystems [27] and RLE thresholds for threatened ecosystems were used here to assess the suitability of globally mapped ecosystems for inclusion in the Critical Habitat data layer under criterion 4. Global scale datasets available for assessment included mangroves [28], saltmarshes [29], ever-wet tropical forests [30], tropical dry forest [31], and tropical montane cloud forest [32, 33]. The RLE is intended to be applied at a finer geographic scale than these global layers. However, as there have been no consistent sub-global assessments of these ecosystem types, each ecosystem dataset was assessed against RLE criteria based on existing scientific literature outlining the global extent and rate of habitat loss of these ecosystem types. We recognise, nevertheless, that there may be substantial regional differences in their vulnerability. For example, past deforestation rates for tropical dry forests range from 2–18% over a 20 year period for different geographical areas [31], and the loss of mangrove forests varies between 3.6% and 1.3% per year in the Americas and Africa, respectively [34]. Integrating regional differences would be valuable but is beyond the scope of this paper. As in all cases, the classification of likely or potential Critical Habitat depends on the alignment with IFC PS6 criteria and scenarios and the spatial resolution of the dataset. Alignment was assessed based on the degree to which the RLE threshold would be met globally. The ecosystem was classed as potential Critical Habitat if regional differences indicated that the RLE threshold would be unlikely to be met consistently across regions.
4. Critical areas for selected species. Only two global datasets at a suitable spatial resolution for selected species were identified: Sea Turtle Nesting Sites and Tiger Conservation Landscapes. The distribution of sea turtle nesting sites [35] was disaggregated based on the species’ IUCN Red List category. All nesting sites were included as potential Critical Habitat under Criteria 3 and 4, and those for Critically Endangered and Endangered species were included as likely Critical Habitat due to alignment with Criteria 1 and 2, respectively. Similarly, we extracted source sites and potential source sites for the Endangered tiger *Panthera tigris* from the Tiger Conservation Landscapes dataset [36]. Source sites are areas that contain concentrations of tigers that have the potential to repopulate larger landscapes and were therefore aligned with Criteria 1. Potential source sites are those where the case for an area’s inclusion as a Source Site is equivocal and more surveys are required [37]. These sites have therefore been included as ‘potential’ Critical Habitat.
5. Threatened species’ ranges. For most species, range maps from the IUCN Red List are not appropriate to include because they represent limits of distribution which can cover vast areas, particularly for wide-ranging species, and are not suitably refined based on an understanding of suitable habitat. However, species that are categorised as highly threatened based on a restricted distribution or population size were considered to be appropriate for inclusion as potential Critical Habitat. Species categorised as Critically Endangered or Endangered under IUCN Red List criterion D (i.e. global population size estimated to number fewer than 50 or 250 mature individuals respectively) were included based on alignment with PS6 Criterion 1. Species categorised as Vulnerable under IUCN Red List criterion D2 (where the population has a very restricted area of occupancy, typically less than 20 km^2^, such that it is prone to the effects of human activities or stochastic events) were included based on alignment with PS6 Criterion 2 [38].

### Coverage and relative composition

**Fig 2:**
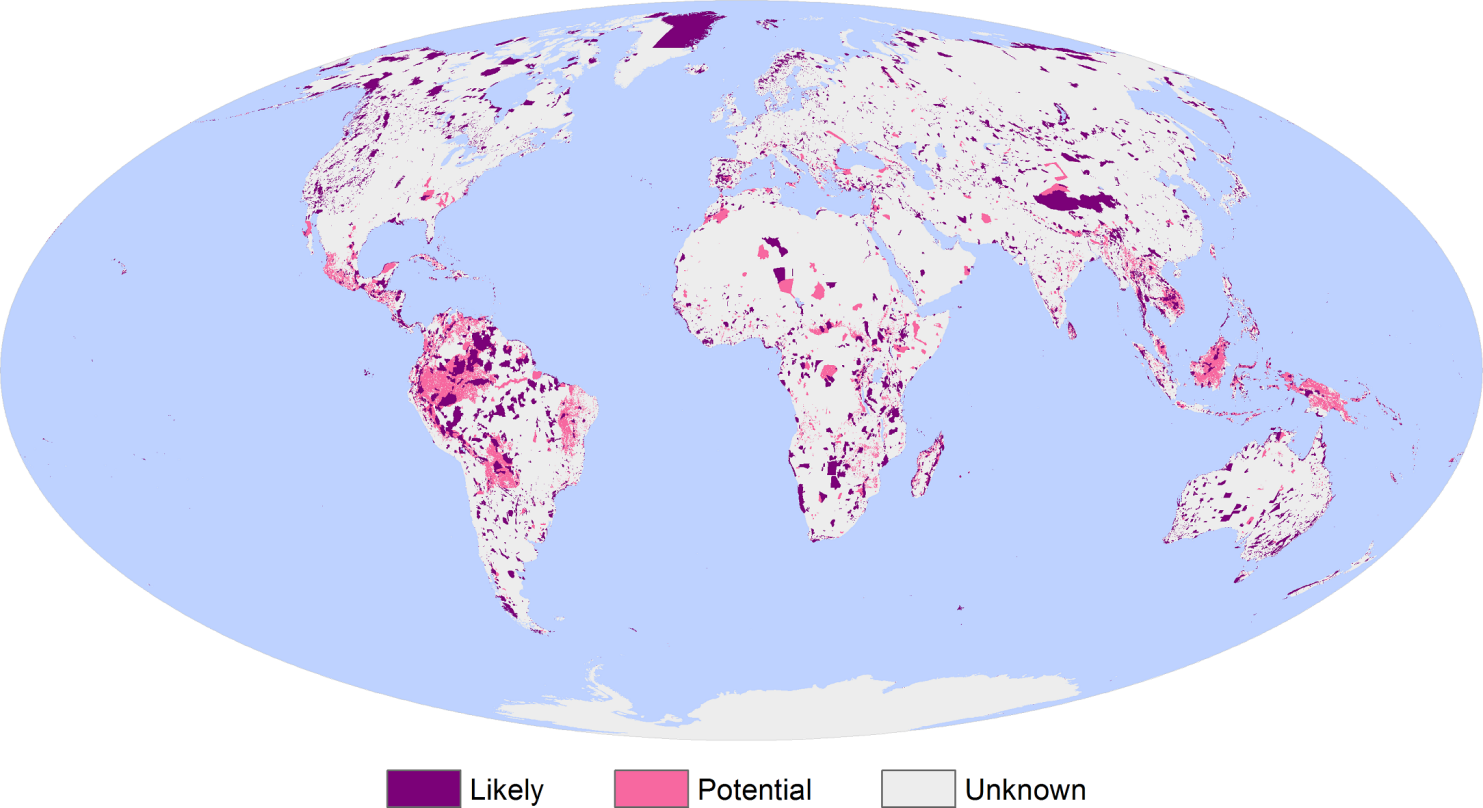
Global screening layer for terrestrial Critical Habitat. Likely and potential Critical Habitat are depicted in purple and pink, respectively. Unknown areas are depicted in dark grey. Marine areas are depicted in blue, and were not assessed. The screening layer is developed as a raster of 1 km grid cell size.

Of the total terrestrial area analysed, 10.1% (14,986 x 10^3^ km^2^) was classified as likely Critical Habitat and 5.1% (7,607 x 10^3^ km^2^) as potential Critical Habitat (Fig 2). The remaining 84.8% (125,794 x 10^3^ km^2^) is classified as unknown, either because the known biodiversity features are not suitably aligned with the Critical Habitat definition or because of a lack of data to make this assessment.

An assessment of the amount of coverage by datasets from individual criteria or scenarios found that scenario B (internationally or nationally recognized areas of high biodiversity value) was the primary driver of area classified as likely Critical Habitat (56% or 8.36 million km^2^; Table 4). Criterion 4 (highly threatened and/or unique ecosystems) was the primary driver of area classified as potential Critical Habitat (78% or 11.92 million km^2^). Together, criteria 1 and 4 and scenario B accounted for approximately one-third of all the area designated as likely and potential Critical Habitat. There were no datasets identified that aligned with criterion 5 or scenario A.

**Table 4:** Surface areas of likely, potential and combined (likely/potential) Critical Habitat (CH) identified under individual criteria and scenarios.

	Likely CH		Potential CH		Likely/Potential CH	
	Area^1^	%	Area^1^	%	Area^1^	%
Criterion 1	6,226	42%	1,032	14%	7,258	32%
Criterion 2	1,458	10%	1,450	19%	2,908	13%
Criterion 3	4,349	29%	4	0%	4,353	19%
Criterion 4	1,542	10%	5,944	78%	7,486	33%
Criterion 5	0	0%	0	0%	0	0%
Scenario A	0	0%	0	0%	0	0%
Scenario B	8,362	56%	0	0%	8,362	37%

^**1**^ Areas: x 10^3^ km^2^

Percentage contribution of each criterion and scenario to the total area of likely, potential or combined Critical Habitat. Total percentage coverage exceeds 100% due to overlapping of areas designated as likely and potential Critical Habitat.

The different biodiversity features assessed also had a varied effect on the classification of Critical Habitat (Table 5). Overall, protected areas and Key Biodiversity Areas triggered the largest areas of Critical Habitat, accounting for 45% (10.16 million km^2^) and 39% (8.73 million km^2^) of combined likely or potential Critical Habitat, respectively. Within these, the largest coverage is recorded for Important Bird and Biodiversity Areas and protected areas under IUCN management categories I-II, which result in 41% and 31% of likely Critical Habitat, respectively. The main contributors to potential Critical Habitat include ever wet tropical forests (28%), tropical dry forests (26%) and Important Bird and Biodiversity Areas (18%). In many cases, more than one biodiversity feature triggered the classification of a single area as Critical Habitat, for example 20% (3.07 million km^2^) of likely Critical Habitat is triggered by three or more data subsets.

**Table 5:** Surface areas of likely, potential and combined (likely/potential) Critical Habitat (CH) triggered by individual biodiversity features.

Biodiversity feature	Likely CH		Potential CH		Likely/ Potential CH	
	Area^1^	%	Area^1^	%	Area^1^	%
Ever wet tropical forests	0	0%	2,143	28%	2,143	9%
Key Biodiversity Areas (all)	7,392	49%	1,333	18%	8,726	39%
Alliance for Zero Extinction sites	642	4%	0	0%	642	3%
Important Bird and Biodiversity Areas	6,173	41%	1,332	18%	7,505	33%
Mangroves	468	3%	0	0%	468	2%
Protected areas (all)	9,169	61%	989	13%	10,158	45%
Natural and mixed World Heritage sites	1,538	10%	0	0%	1,538	7%
Ramsar sites	1,074	7%	0	0%	1,074	5%
Irreplaceable protected areas	1,145	8%	0	0%	1,145	5%
IUCN management categories Ia, Ib, II	6,964	46%	0	0%	6,964	31%
≥10% of CR/EN species ranges overlap	0	0%	931	12%	931	4%
≥95% endemic, restricted-range ranges overlap	0	0%	233	3%	233	1%
Saltmarshes	176	1%	0	0%	176	1%
Sea turtle nesting sites	93	1%	4	0%	97	0%
Threatened species (D/D2)	205	1%	816	11%	1,020	5%
Tiger Conservation Landscapes	99	1%	27	0%	126	1%
Tropical dry forests	0	0%	1,959	26%	1,959	9%
Tropical montane cloud forests	0	0%	931	12%	931	4%

^**1**^ Areas: x 10^3^ km^2^

Percentage figures report the contribution of each feature to the total area of likely, potential or combined Critical Habitats. Total percentage coverage exceeds 100% due to overlapping of areas designated as likely and potential Critical Habitat.

## Discussion

### Current and future datasets for Critical Habitat

This study presents a single terrestrial layer derived from 12 biodiversity datasets that align with one or more of the criteria defined by IFC PS6 for Critical Habitat or one of two scenarios based on the PS6 standard and guidance notes. The final Critical Habitat screening layer covers 15% of the terrestrial surface. While the distribution of potential or likely Critical Habitat varies across countries and regions, this pattern is in part driven by the availability of data and therefore cannot accurately reflect the actual distribution of Critical habitat.

The screening layer has gaps, as reliable and up-to-date spatial information is not available across all taxonomic groups and ecosystem types. For example, of an estimated 8.7 million species on earth [39], only ca. 85,000 species have been assessed for the IUCN Red List of Threatened Species. Furthermore, where they exist, range maps for most species show distributional boundaries rather than known occupancy owing to a lack of sufficiently high-resolution data on presence or confirmed absence. Likewise, global spatial data on ecosystems often focus on more economically valuable ecosystems, or those that can be mapped using remote sensing, such as forests [40] and wetlands [41]. For nearly all countries worldwide, KBAs have been identified for birds (IBAs) and for highly threatened and range-restricted vertebrates, corals and conifers (AZEs). While KBAs have been identified for other taxonomic groups in many countries, coverage is far from complete [42]. The recent adoption of a new global standard for the identification of KBAs [25], and the launch of the *World Database of Key Biodiversity Areas* [24] should help to expand rapidly the comprehensiveness of the global KBA inventory. While there are a large number of national and local survey efforts underway to map species and ecosystems, compiling these data at the global level remains a significant task.

Data availability varied across the IFC criteria. Specifically, Criterion 1 (Habitats of significant importance to Critically Endangered and/or Endangered species), Criterion 2 (Habitats of significant importance to endemic and/or range restricted species) and Criterion 3 (Habitats supporting globally significant concentrations of migratory and/or congregatory species) were particularly influential in the final screening layer due to the availability of large polygon-based spatial datasets (i.e. KBAs and protected areas) that aligned well with the IFC criteria.

Criterion 4 (Highly threatened or unique ecosystems) was represented by specific ecosystems (e.g. saltmarshes, mangroves, or tropical dry, montane or ever-wet forests). Expert review of the forest datasets concluded regional variations in their threatened status and/or relatively low levels of certainty in relation to on-ground presence of these mapped ecosystems. Consequently, this resulted in the attribution of potential (rather than likely) Critical Habitat status to these layers (Table 3). Data certainty is higher for datasets on the global distribution of saltmarshes and mangroves, resulting in a classification as likely Critical Habitat. These were compiled based on occurrence datasets (saltmarsh) and Landsat imagery validated through distribution data and published literature (mangroves). Criterion 5 (Areas associated with key evolutionary processes) had no available data as a result of the difficulty associated with mapping these areas.

There are a large number of broad-scale priority-setting approaches that identify regions of the world based on varying degrees of irreplaceability and/or vulnerability. These include Biodiversity Hotspots [43], Endemic Bird Areas [44], Intact Forest Landscapes [45], Centres of Plant Diversity [46], Crisis Ecoregions [47], Megadiversity Countries [48], Last of the Wild [49], High-Biodiversity Wilderness Areas [50], and Global 200 ecoregions [51]. While these areas are aligned with the definition of Critical Habitat (S4 Table) they are spatially extensive and not sufficiently refined to indicate presence on the ground and are therefore excluded from this screening layer. However, they do provide important background context that could support Critical Habitat assessments and inform their interpretation.

This screening layer is envisaged to evolve as new global datasets become available. There are a number of different types of data which could, with some further development, be included in subsequent iterations of this analysis. Of the 36 datasets that were considered for inclusion in this study, 25 were excluded as they were too coarse to indicate presence of the features at a resolution suitable for project planning (e.g. IUCN range data and a number of datasets that are based on these, Crisis Ecoregions, Biodiversity Hotspots); were considered insufficiently aligned with Critical Habitat criteria (e.g. a number of subsets of the Global Lakes and Wetlands database, and datasets of physical features such as mountains and islands); or were not finalised or publicly available (e.g. High Conservation Value areas, and Important Plant Areas [52] (S2 Table).

Of datasets currently under development, the IUCN Red List of Ecosystems (RLE) is particularly noteworthy in the context of this assessment. As yet the RLE is only represented by 20 case studies spread across the world [53]. Although it is expected to be several years before global maps are available, national maps will be released gradually over the coming years. The RLE is specifically referred to within the IFC criteria and in later years will be the principal global dataset that aligns with Criterion 4 on highly threatened and/or unique ecosystems. Other datasets that will likely become available and inform future iterations of this screening layer include refined range data based on extent of suitable habitat for threatened and endemic species, and globally collated assessment data for High Conservation Value areas. It is therefore expected that over time the screening layer will become more comprehensive both geographically as well as in its coverage of taxonomic groups and ecosystem types.

### Interpretation and use of the Critical Habitat screening layer

The screening layer of Critical Habitat can provide information for companies in the early stages of project development by highlighting areas of potential or likely Critical Habitat presence. By providing information on the underlying biodiversity features that trigger classification, this screening layer can inform subsequent more detailed Critical Habitat assessments by companies and support the evaluation of biodiversity risk of proposed operations by finance institutions.

However it is important to recognise the limitations of a data layer based on currently available, global spatial data. In particular, the classification of an area as ‘unknown’ in this assessment can include areas of Critical Habitat for which there are no available global data or areas without the necessary biodiversity values to trigger a Critical Habitat designation. There are many elements of the Critical Habitat definition for which no appropriate global data are available. In addition, the resolution, age, and completeness of the datasets create the potential for omissions (where datasets omit the presence of the biodiversity feature) and commission (where a biodiversity feature is reported as present but is actually absent). Definitive identification of Critical Habitat that is needed prior to planning projects and mitigation measures will require finer scale data (e.g. records of threatened species at particular locations, which are increasingly available through citizen science repositories) combined with in-situ biodiversity surveys by qualified assessors to confirm or dismiss the presence of Critical Habitat and more accurately define boundaries. Once confirmed and defined and operators decide to continue, IFC PS6 requires those values for which it is designated to be enhanced to deliver a net gain of biodiversity and therefore robust mitigation measures are required to achieve this.

As presented in this paper, a static compound layer means that some areas of Critical Habitat may not be visible when viewed at a global scale and it precludes the ability to interrogate the layer to identify the underlying features triggering classification. The utility and impact of this work would be increased by making the layer available through an interactive platform, such as the Integrated Biodiversity Assessment Tool (IBAT, [12]) where users could zoom in to specific areas of interest and add contextual layers by providing access to relevant information on biodiversity features not included in the screening layer. For example, protected areas which are not all incorporated into the Critical Habitat screening layer are still subject to a set of requirements on the basis of their legal designation and / or international importance [6]. Equally some of the broad-scale priority-setting approaches such as Endemic Bird Areas and Biodiversity Hotspots referred to in the earlier section that are aligned with the Critical Habitat definition (S4 Table) but excluded on the basis of their spatial resolution, are included in IBAT and would add important context to this screening layer. Tools such as IBAT are regularly updated as new data become available and data users are therefore accustomed to the evolving nature of these datasets.

Using this layer in synergy with the marine version [18] will provide insight regarding global Critical Habitat across all realms. Results presented in Martin et al. [18] revealed a lower proportional coverage of Critical Habitat in the marine realm, with 1.6% and 2.1% of the area classified as likely and potential Critical Habitat. This is due to the lower number of datasets available for the marine realm, particularly in areas beyond national jurisdiction and the deep seas [54; 55] as well as the spatial resolutions of those datasets. This can be seen in differences between the spatial properties (geometries) of the features used in the two layers; the terrestrial features are generally better resolved, showing greater detail around feature boundaries.

This study provides a methodology to integrate a number of global biodiversity datasets and make them accessible and relevant to business at a crucial stage in the project life cycle. By tailoring the layer to the biodiversity specificities of a particular standard to which business are complying (IFC PS6) there is a direct and much needed application for this work. This study has highlighted a range of opportunities for expanding and improving the data used within this screening layer and the next step is to operationalise this as an accessible, evolving and updateable data product aimed at supporting improvements to biodiversity screening and management.

## Supporting information

S1 Table Biodiversity features included in the screening layer for the identification of Critical Habitat. Includes information on which element of the PS6 Critical Habitat they align with, the dataset used, their classification as likely or potential Critical Habitat and justification based on the degree of alignment with the PS6 definition and the certainty with which the data indicates presence of the biodiversity feature at a 1 km^2^ resolution.
(DOCX)

S2 Table Further datasets considered and justification for exclusion. Includes information on which element of the PS6 Critical Habitat they align with, the dataset investigated and the reason it was excluded from the global screening layer.
(DOCX)

S3 Table Proposed alignment between the KBA criteria in the new global standard for the identification of KBAs (IUCN 2016) and IFC Critical Habitat (CH) criteria. (DOCX)

S4 Table Relevance of regional-scale designations to Critical Habitat criteria. (DOCX)

S1 Fig Global screening layer (1x1 km raster) for terrestrial Critical Habitat. A GIS dataset of the terrestrial Critical Habitat screening layer is available on request for research and conservation purposes from information@unep-wcmc.org.
(DOCX)

## References

[1] TEEB. The Economics of Ecosystems and Biodiversity: Mainstreaming the Economics of Nature: A Synthesis of the Approach, Conclusions and Recommendations of TEEB. UNEP; 2010.

[2] World Conservation Congress 2016 resolution 67.Best practice for Industrial scale activities. IUCN (2016). IUCN Resolutions, Recommendations and other Decisions. Gland, Switzerland: IUCN. 106pp.

[3] Hanson C, Ranganathan J, Iceland C, Finisdore J. The Corporate Ecosystem Services Review: Guidelines for Identifying Business Risks and Opportunities Arising from Ecosystem Change: Version 2.0. Washington, DC: World Resources Institute; 2012.

[4] UNEP-WCMC. Review of the Biodiversity Requirements of Standards and Certification Schemes: A Snapshot of Current Practice. Montreal, Canada: Secretariat of the Convention on Biological Diversity; 2011. Report No.: CBD Technical Series No. 63.

[5] Morgera E. From corporate social responsibility to accountability mechanisms: The Role of the Convention on Biological Diversity. 2012 p. 321–54. Report No.: Edinburgh School of Law Research Paper Series 06.

[6] IFC. Performance Standard 6: Biodiversity Conservation and Sustainable Management of Living Natural Resources. International Finance Corporation; 2012 Available from: www.ifc.org

[7] CBD. Decisions of the Eleventh Meeting of the Conference of the Parties to the Convention on Biological Diversity. 2012. Available from: https://www.cbd.int/decisions/cop/?m=cop-11

[8] Equator Principles. The Equator Principles III. June 2013. Available from: www.equator-principles.com

[9] Equator Principles Secretariat. The Equator Principles. About the Equator Principles. 2011 [cited 2017 Feb 28]. Available from: http://www.equator-principles.com/index.php/ep3/38-about/about/195

[10] IFC. Guidance Note 6: Biodiversity Conservation and Sustainable Management of Living Natural Resources. International Finance Corporation; 2012 Available from: www.ifc.org

[11] Oyu Tolgoi Project Critical Habitat Assessment: IFC Performance Standard 6/EBRD Performance Requirement 6. The Biodiversity Consultancy, Fauna & Flora International; 2012 8 Available from: http://ot.mn/media/ot/content/page_content/commitments/ESIA/1_ESIA/Biodiversity_Appendices/ESIA_BA2_Critical_Habitat_Assessment.pdf

[12] BirdLife International, Conservation International, IUCN, UNEP-WCMC. Integrated Biodiversity Assessment Tool (IBAT). IBAT for Business Available from: www.ibatforbusiness.org

[13] Willis KJ, Jeffers ES, Tovar C, Long PR, Caithness N, Smit MGD, et al Determining the ecological value of landscapes beyond protected areas. Biol Conserv. 2012;147(1):3–12.

[14] Ball I, Watts H, Possingham M. Marxan and Relatives: Software for Spatial Conservation Prioritisation In: Moilanen A, Wilson KA, Possingham H, editors. Spatial Conservation Prioritisation: Quantitative Methods and Computational Tools. Oxford (UK): Oxford University Press; 2009 p. 185–95.

[15] Merrifield MS, Mcclintock W, Burt C, Fox E, Serpa P, Steinback C, et al MarineMap: A web-based platform for collaborative marine protected area planning. Ocean Coast Manag. 2013;74:67–76.

[16] Kareiva P, Tallis H, Ricketts TH, Daily GC, Polasky S. Natural Capital: Theory and Practice of Mapping Ecosystem Services. OUP Oxford; 2011. 392 p.

[17] Villa F, Bagstad KJ, Voigt B, Johnson GW, Portela R, Honzák M, et al A methodology for adaptable and robust ecosystem services assessment. PLoS One. 2014 3 13;9(3):e91001 doi: 10.1371/journal.pone.0091001 24625496 10.1371/journal.pone.0091001 PMC3953216

[18] Martin CS, Tolley MJ, Farmer E, Mcowen CJ, Geffert JL, Scharlemann JPW, et al A global map to aid the identification and screening of critical habitat for marine industries. Mar Policy. 2015;53:45–53.

[19] World Bank. Environmental and Social Framework. Setting Environmental and Social Standards for Investment Project Financing 2016 8.

[20] Wessel P, Smith WHF. GSHHG. A Global Self-consistent, Hierarchical, High-resolution Geography Database. Version 2.2.2. 2013. Available from: https://www.soest.hawaii.edu/pwessel/gshhg/

[21] Le Saout S, Hoffmann M, Shi Y, Hughes A, Bernard C, Brooks TM, et al Conservation. Protected areas and effective biodiversity conservation. Science. 2013 11 15;342(6160):803–5. doi: 10.1126/science.1239268 24233709 10.1126/science.1239268

[22] UNEP-WCMC and IUCN (2017), The World Database on Protected Areas (WDPA), [February 2017 version downloaded from IBAT], Cambridge, UK: UNEP-WCMC and IUCN Available at: www.protectedplanet.net

[23] IUCN. The IUCN Red List of Threatened Species. Version 2016–1. Available from: www.iucnredlist.org 10.3897/BDJ.4.e10356 PMC5018116 27660524

[24] BirdLife International. World Database of Key Biodiversity Areas. Developed by the KBA Partnership. 2016. Available from: www.keybiodiversityareas.org

[25] IUCN. A Global Standard for the Identification of Key Biodiversity Areas. Version 1.0. 2016 Mar. Available from: https://portals.iucn.org/library/sites/library/files/documents/Rep-2016-005.pdf

[26] Langhammer PF, Bakarr MI, Bennun LA, Brooks TM, Clay RP, Darwall W, et al Identification and gap analysis of key biodiversity areas: targets for comprehensive protected area systems Gland, Switzerland: IUCN; 2007.

[27] Keith DA, Rodríguez JP, Rodríguez-Clark KM, Nicholson E, Aapala K, Alonso A, et al Scientific foundations for an IUCN Red List of ecosystems. PLoS One. 2013 5 8;8(5):e62111 doi: 10.1371/journal.pone.0062111 23667454 10.1371/journal.pone.0062111 PMC3648534

[28] Giri C, Ochieng E, Tieszen LL, Zhu Z, Singh A, Loveland T, et al Status and distribution of mangrove forests of the world using earth observation satellite data (version 1.3, updated by UNEP-WCMC). Glob Ecol Biogeogr. 20:154–9.

[29] UNEP-WCMC, Conservation International, The Nature Conservancy. Global distribution of saltmarsh (ver. 4.0). Cambridge (UK): UNEP World Conservation Monitoring Centre; Available from: http://data.unep-wcmc.org/datasets/43

[30] Underwood EC, Olson D, Hollander AD, Quinn JF. Ever-wet tropical forests as biodiversity refuges. Nat Clim Chang. 2014 8 27;4(9):740–1.

[31] Miles L, Newton AC, DeFries RS. A global overview of the conservation status of tropical dry forests. Journal of Biogeography. 2006; Available from: http://onlinelibrary.wiley.com/doi/10.1111/j.1365-2699.2005.01424.x/full

[32] Bubb P, May IA, Miles L, Sayer J. Cloud forest agenda UNEP World Conservation Monitoring Centre; 2004.

[33] Aldrich et al. A Global Directory of Tropical Montane Cloud Forests. 1997.

[34] Vaiela I, Bowen JL, York JK. Mangrove Forests: One of the World’s Threatened Major Tropical Environ ments. BioScience. 2001 10;51(10).

[35] UNEP-WCMC. Global distribution of sea turtle nesting sites (version 1.1) Cambridge (UK): UNEP World Conservation Monitoring Centre; 1999 [cited 2016 Jun 1]. Available from: http://data.unep-wcmc.org/datasets/22

[36] Walston J, Robinson JG, Bennett EL, Breitenmoser U, da Fonseca GAB, Goodrich J, et al Bringing the tiger back from the brink-the six percent solution. PLoS Biol. 2010 9 14;8(9). Available from: http://dx.doi.org/10.1371/journal.pbio.1000485 10.1371/journal.pbio.1000485 PMC2939024 20856904

[37] Walston J., Karanth K.U., and Stokes E.J. 2010 Avoiding the Unthinkable: What Will it Cost to Prevent Tigers Becoming Extinct in the Wild? Wildlife Conservation Society, New York.

[38] IUCN. IUCN Red List Categories and Criteria. Version 3.1. Second edition. www.iucnredlist.org/technical-documents/categories-and-criteria; 2001.

[39] Mora C, Tittensor DP, Adl S, Simpson AGB, Worm B (2011) How Many Species Are There on Earth and in the Ocean? PLoS Biol 9(8): e1001127 doi: 10.1371/journal.pbio.1001127 21886479 10.1371/journal.pbio.1001127 PMC3160336

[40] Hansen MC, Potapov PV, Moore R, Hancher M, Turubanova SA, Tyukavina A, et al High-Resolution Global Maps of 21st-Century Forest Cover Change. Science. 2013 11 15;342(6160):850–3. doi: 10.1126/science.1244693 24233722 10.1126/science.1244693

[41] Dixon MJR, Loh J, Davidson NC, Beltrame C, Freeman R, Walpole M. Tracking global change in ecosystem area: The Wetland Extent Trends index. Biol Conserv. 2016/1;193:27

[42] Foster MN, Brooks TM, Cuttelod A, De Silva N, Fishpool LDC, Radford EA, et al. The identification of sites of biodiversity conservation significance: progress with the application of a global standard. Available from: http://threatenedtaxa.org/index.php/JoTT/article/download/779/1392

[43] Conservation International. Biodiversity Hotspots Revisited. 2011. Available from: www.biodiversityhotspots.org/xp/Hotspots/resources/maps.xml

[44] BirdLife International. Endemic Bird Areas. 1998.

[45] Potapov P, Yaroshenko A, Turubanova S, Dubinin M, Laestadius L, Thies C, et al Mapping the world’s intact forest landscapes by remote sensing. Ecol Soc. 2008;13(2). Available from: http://www.ecologyandsociety.org/vol13/iss2/art51/main.html

[46] UNEP-WCMC. Centres of Plant Diversity. Version 1.0 (digital reproduction of Centres of Plant Diversity, eds S.D. Davis, V.H. Heywood & A.C. Hamilton, WWF and IUCN, Gland, Switzerland, 1994–7). 2013.

[47] Hoekstra JM, Boucher TM, Ricketts TH, Roberts C. Confronting a biome crisis: global disparities of habitat loss and protection. Ecol Lett. 2005 1 1;8(1):23–9.

[48] Mittermeier RA, Robles-Gil P, Mittermeier CG, editors. Megadiversity: Earth’s biologically wealthiest nations. CEMEX/Agrupacion Sierra Madre; 1997.

[49] Sanderson EW, Jaiteh M, Levy MA, Redford KH, Wannebo AV, Woolmer G. The human footprint and the last of the wild: the human footprint is a global map of human influence on the land surface, which suggests that human beings are stewards of nature, whether we like it or not. Bioscience. 2002;52(10):891–904.

[50] Mittermeier RA, Mittermeier CG, Brooks TM, Pilgrim JD, Konstant WR, da Fonseca GAB, et al Wilderness and biodiversity conservation. Proc Natl Acad Sci U S A. 2003 9 2;100(18):10309–13. doi: 10.1073/pnas.1732458100 12930898 10.1073/pnas.1732458100 PMC193557

[51] Brooks TM, Mittermeier RA, da Fonseca GAB, Gerlach J, Hoffmann M, Lamoreux JF, et al Global biodiversity conservation priorities. Science. 2006 7 7;313(5783):58–61. doi: 10.1126/science.1127609 16825561 10.1126/science.1127609

[52] Plantlife International (2004) Identifying and Protecting the World’s Most Important Plant Areas: A Guide to Implementing Target 5 of the Global Strategy for Plant Conservation. Salisbury, UK: Plantlife International

[53] IUCN. Regional Assessments. IUCN Red List of Ecosystems. [cited 2017 Feb 28]. Available from: www.iucnrle.org/assessments/

[54] Costello MJ, Coll M, Danovaro R, Halpin P, Ojaveer H, Miloslavich P. A census of marine biodiversity knowledge, resources, and future challenges. PLoS One. 2010 8 2;5(8):e12110 doi: 10.1371/journal.pone.0012110 20689850 10.1371/journal.pone.0012110 PMC2914025

[55] Webb TJ, Vanden Berghe E, O’Dor R. Biodiversity’s big wet secret: the global distribution of marine biological records reveals chronic under-exploration of the deep pelagic ocean. PLoS One. 2010 8 2;5(8):e10223 doi: 10.1371/journal.pone.0010223 20689845 10.1371/journal.pone.0010223 PMC2914017

