# Supplementary material for: Global screening for Critical Habitat in the terrestrial realm
Source: PLoS One. 2018 Mar 22;13(3):e0193102. doi: 10.1371/journal.pone.0193102 (PMC5863962; doi:10.1371/journal.pone.0193102)
Supplement: S1 Table — Includes information on which element of the PS6 Critical Habitat they align with, the dataset used, their classification as likely or potential Critical Habitat and justification based on the degree of alignment with the PS6 definition and the certainty with which the data indicates presence of the biodiversity feature at a 1 km2 resolution. (DOCX) [file pone.0193102.s001.docx]

**S1 Table. Biodiversity features included in the screening layer for the identification of candidate Critical Habitat.** Includes information on which element of the PS6 Critical Habitat they align with, the dataset used, their classification as likely or potential Critical Habitat and justification based on the degree of alignment with the PS6 definition and the certainty with which the data indicates presence of the biodiversity feature at a 1km^2^ resolution.

⚫: High (Data certainty or Alignment) 🞆: Medium (Data certainty or alignment)

| **Critical Habitat description** | **Relevant**  **biodiversity feature** | **Dataset(s)**  **and reference** | **Data certainty /prescem** | **Alignment** | **Justification** | **CH classification^[[1]](#footnote-1)^** |
| --- | --- | --- | --- | --- | --- | --- |
| **Criterion 1: Habitats of significant importance to Critically Endangered (CE) and/or Endangered (EN) species^[[2]](#footnote-2)^** | | | | | | |
| Habitat required to sustain ≥ 10% of the global population of an IUCN Red-listed CR or EN species where there are known, regular occurrences of the species and where that habitat could be considered a discrete management unit for that species | Alliance for Zero Extinction (AZE) sites | World Database of Key Biodiversity Areas [24] | ⚫ | ⚫ | Contains at least 95% of the known population of a CR or EN species. (NOTE: These data are aggregated with the AZEs included under Criterion 2 and 3) | **Likely** |
|  | Protected areas | World Database on Protected Areas [22] and Red List of Threatened Species [23] | ⚫ | 🞆 | Protected areas that overlap with ≥10% of the range of one or more CR or EN species. As this is a proxy measure for % population contained within a protected area, there is less certainty that these sites will align and they are therefore included as potential Critical Habitat. | **Potential** |
| Habitat with known, regular occurrences of CR or EN species where that habitat is one of 10 or fewer discrete management sites globally for that species | Important Bird and Biodiversity Areas (IBAs) triggered by species that occupy 10 or fewer sites | World Database of Key Biodiversity Areas [24] | ⚫ | ⚫ | Sites triggered by CR or EN species that occupy 10 sites or fewer. | **Likely** |
| Habitat that supports the regular occurrence of a single individual of an IUCN Red-listed CR species and/or habitat containing regionally-important concentrations of an IUCN Red-listed EN species where that habitat could be considered a discrete management unit for that species | Key Biodiversity Areas (KBAs) designated under criterion 1: vulnerability | World Database of Key Biodiversity Areas [24], including data on trigger species | ⚫ | ⚫ | Sites contain regular occurrence of (i) a single individual of a CR or EN species at the site and/or (ii) 30 individuals or 10 pairs of VU species. The number of individuals of EN trigger species at particular KBAs has not been assessed, but for the large majority of sites is expected to qualify as ‘regionally significant’: hence these sites are included as likely Critical Habitat. Sites triggered by VU species are not included. | **Likely** |
|  | IBAs designated under criterion A1: Globally [threatened species](http://biodiversitya-z.org/content/threatened-species) | World Database of Key Biodiversity Areas [24] including trigger species data. | ⚫ | ⚫ | Sites regularly hold significant numbers of a CR, EN or VU species. For EN species, this is taken to qualify their presence as ‘regionally significant’, so these sites are included as likely Critical Habitat. IBAs triggered by VU species are not included. | **Likely** |
|  | Ramsar sites designated under criterion 2 | World Database on Protected Areas [22] | ⚫ | ⚫ | Wetlands that support vulnerable, endangered, or critically endangered species or threatened ecological communities. As per GN57 all Ramsar sites will be likely Critical Habitat, however under this criterion only those triggered by CR and/or EN species would be likely. | **Likely** |
|  | Turtle nesting sites | Global distribution of sea turtle nesting sites [35] | ⚫ | ⚫ | Nesting sites for 3 CR and 2 EN species are included. Nesting sites for EN species are expected to qualify as ‘regionally significant’ in the large majority of cases, hence these sites are included as likely Critical Habitat. | **Likely** |
|  | IUCN range maps for D species (CR and EN only) | Spatial data for the Red List of Threatened Species [23, 48] | ⚫ | ⚫  CR  🞆  EN | CR or EN species listed under IUCN Red List criterion D (i.e. owing to a global population size estimated to number fewer than 50 or 250 mature individuals respectively). These species occupy very small areas where there is a high likelihood of presence at a local scale. Those triggered by CR species are included as likely Critical Habitat, and those triggered by EN species as potential Critical Habitat as it is unknown if there would be regionally important concentrations within these range distributions. | **Likely** for CR species*.*  **Potential** for EN species. |
| Habitat of significant importance to CR or EN species that are wide-ranging and/or whose population distribution is not well understood and where the loss of such a habitat could potentially impact the long-term survivability of the species | Tiger source sites | Tiger Conservation Landscapes [36] | ⚫ | ⚫  🞆 | Tiger source sites have a high density of the Endangered (EN) tiger and the potential to maintain a demographically viable cluster of >25 breeding females. These 42 sites contain 70% of all remaining tigers and are considered to qualify as regionally significant. Potential source sites where there is less certainty that all the criteria are met are included as potential Critical Habitat. | **Likely** (source sites)  **Potential** (potential source sites) |
| As appropriate, habitat containing nationally/regionally-important concentrations of an EN, CR or equivalent national/regional listing | - | - | - | - | - | - |
| **Criterion 2: Habitats of significant importance to endemic and/or restricted-range species** | | | | | | |
| Habitat known to sustain ≥ 95% of the global population of an endemic or restricted-range species where that habitat could be considered a discrete management unit for that species (e.g., a single-site endemic) | AZEs | World Database of Key Biodiversity Areas [24] | ⚫ | ⚫ | Sites contain at least 95% of the known population of a CR or EN species for endemic or restricted range species. (NOTE: These data are aggregated with the AZEs included under Criterion 1 and 3). | **Likely** |
|  | Protected areas | World Database on Protected Areas [22] and Red List of Threatened Species [23] | ⚫ | 🞆 | Protected Areas overlapping with ≥95% of the global range of endemic or restricted-range species. As this is a proxy measure for % population contained within a protected area, there is less certainty that these sites will align and they are therefore included as potential Critical Habitat. | **Potential** |
| Habitat known to sustain ≥ 1% but < 95% of the global population of an endemic or restricted-range species where that habitat could be considered a discrete management unit for that species, where adequate data are available and/or based on expert judgment | KBAs designated under criterion 2A | World Database of Key Biodiversity Areas [24] | ⚫ | ⚫ | Site holds 5% of global population for species with a global range < 50,000 km^2^ (at any stage of the species’ lifecycle). | **Likely** |
|  | IBAs designated under criterion A2 | World Database of Key Biodiversity Areas [24] | ⚫ | 🞆 | Site known or thought to hold a significant component of a group of species whose breeding distributions define an Endemic Bird Area (EBA: two or more species of range < 50,000km^2^ occur together) or Secondary Area (SA: supports ≥1 restricted-range species, but <2 species are entirely confined to it). It is uncertain whether such sites contain >1% of a trigger species’ global population and they are therefore included as potential Critical Habitat. | **Potential** |
|  | IUCN range maps for D2 listed species | Spatial data for the Red List of Threatened Species [23] | ⚫ | ⚫ | Distributions of species categorised as VU under IUCN criterion D2 where the population has a very restricted area of occupancy, typically less than 20km^2^ or fewer than five locations, such that it is prone to the effects of human activities or stochastic events. | **Likely** |
| **Criterion 3: Habitats supporting globally significant concentrations of migratory species and/or congregatory species** | | | | | | |
| Habitat known to sustain, on a cyclical or otherwise regular basis, ≥ 95% of the global population of a migratory or congregatory species at any point of the species’ life-cycle where that habitat could be considered a discrete management unit for that species | AZEs for congregatory and migratory species | World Database of Key Biodiversity Areas [24] | ⚫ | ⚫ | Site supports at least 95% of the known population of a CR or EN migratory or congregatory species) at some point in their life-cycle. (NOTE: These data are aggregated with the AZEs included under Criterion 1 and 2). | **Likely** |
|  | IBAs under criterion A4 | World Database of Key Biodiversity Areas [24] | ⚫ | ⚫ | Sites supports on a regular basis, >1% of the global population of a congregatory species. | **Likely** |
| Habitat known to sustain, on a cyclical or otherwise regular basis, ≥ 1% but < 95% of the global population of a migratory or congregatory species at any point of the species’ life-cycle and where that habitat could be considered a discrete management unit for that species, where adequate data are available and/or based on expert judgment | KBAs designated under criterion 2C | World Database of Key Biodiversity Areas [24] | ⚫ | ⚫ | Sites that contain globally significant [congregations](http://biodiversitya-z.org/content/congregatory-species) containing 1% of global population of species seasonally occurring at the site. | **Likely** |
|  | Turtle nesting sites | Global distribution of Sea Turtle Nesting Sites [35] | ⚫ | 🞆 | All turtle nesting sites are sites of turtle congregation for breeding. It is uncertain whether any particular site contains >1% of the global population and therefore they are included as potential Critical Habitat. | **Potential** |
|  | Ramsar sites designated under criteria 4,7,8 and 9 | World Database on Protected Areas [22] | ⚫ | 🞆 | Congregations of non-avian species. It is uncertain whether any particular site contains >1% global population of the species in question, and they are included as potential Critical habitat under this criterion. However all Ramsar sites will be considered likely Critical Habitat under scenario B based on GN57. | **Potential** |
| For birds, habitat that meets BirdLife International’s criterion A4 for congregations and/or Ramsar Criteria 5 or 6 for Identifying Wetlands of International Importance | IBAs designated under criterion A4 | World Database of Key Biodiversity Areas [24] | ⚫ | ⚫ | Congregation sites that have a direct mention in GN6. | **Likely** |
|  | Ramsar sites designated under criteria 5 and 6 | World Database on Protected Areas [22] | ⚫ | ⚫ | Wetlands that regularly support 20,000 or more waterbirds (Cr 5) or 1% of the individuals in a population of one species or subspecies of water birds (Cr 6). These have a direct mention in GN6. | **Likely** |
| For species with large but clumped distributions, a provisional threshold is set at ≥ 5% of the global population for both terrestrial and marine species | KBAs designated under criterion 2B | World Database of Key Biodiversity Areas [24] | ⚫ | ⚫ | Sites with species with large but clumped distributions containing 5% of global population at the site. | **Likely** |
| Source sites that contribute ≥ 1% of the global population of recruits | KBAs designated under criterion 2D | World Database of Key Biodiversity Areas [24] | ⚫ | ⚫ | Sites with globally significant source populations. | **Likely** |
| **Criterion 4: Highly threatened and/or unique ecosystems^[[3]](#footnote-3)^** | | | | | | |
| Highly threatened or unique ecosystems are those:   - that are at risk of significantly decreasing in area or quality; - with a small spatial extent; and/or - containing unique assemblages of species including assemblages or concentrations of biome-restricted species | Ramsar sites designated under criteria 1&3 | World Database on Protected Areas [22] | ⚫ | ⚫ | Wetlands that contain a representative, rare or unique example of a natural or near-natural wetland type found within the appropriate biogeographic region (Cr1), and wetlands that support populations of plant and/or animal species important for maintaining the biological diversity of a particular biogeographic region (Cr3). | **Likely** |
|  | KBAs designated under criterion 2E | World Database of Key Biodiversity Areas [24] | 🞆 | ⚫ | Sites containing bioregionally restricted assemblages. There is some subjectivity around the identification of these areas and they are therefore included as potential Critical Habitat. | **Potential** |
|  | IBAs designated under criterion A3 | World Database of Key Biodiversity Areas [24] | 🞆 | ⚫ | Sites containing biome-restricted species. There is some subjectivity around the identification of these areas and they are therefore included as potential Critical Habitat. | **Potential** |
|  | Saltmarshes | Global Distribution of Saltmarsh [29] | ⚫ | ⚫ | Ecosystems considered threatened in accordance with the RLE threshold for VU ecosystems [49] based on the rate of loss of saltmarshes of around 1-2% per year [50] and the historic loss estimated at between 25% and 50% of their global historical coverage [51, 52]. | **Likely** |
|  | Mangroves | Global Distribution of Mangroves [28] | ⚫ | ⚫ | Ecosystems considered threatened in accordance with the RLE threshold for VU ecosystems [49] based on 20% of the total area of mangroves being lost between 1980 and 2005 [52,53] and the continued decline at an estimated rate of 1-2% annually [54]. | **Likely** |
|  | Turtle nesting sites | Global Distribution of Sea Turtle Nesting Sites [35] | ⚫ | 🞆 | Turtle nesting beaches have shown a 20% loss of historic extent and 50% of the remaining nesting sites have been reduced to dangerously low populations [55]. Although we do not know the total area of nesting sites lost, based on the number of sites, this is approaching the RLE threshold for vulnerable ecosystems and they are therefore included as potential Critical Habitat. | **Potential** |
|  | Tropical Montane Cloud forest | Global directory of tropical montane cloud forests [32], [33] | ⚫ | ⚫ | Ecosystems considered threatened in accordance with the RLE threshold for VU ecosystems (Bland et al., 2016) based on a historic loss of original cloud forest cover by 55% [56]. | **Likely** |
|  | Ever wet tropical forests | The Ever-wet Tropical Forest zones are identified within the tropical and subtropical moist broadleaf forest biome [71], where forest cover is ≥75% (based on MODIS data) ([DiMiceli et al. 2011](#_ENREF_2)) as illustrated in [30] | 🞆 | ⚫ | Ecosystems considered threatened in accordance with the RLE threshold for VU ecosystems (Bland et al., 2016) based on an estimated loss of intact natural habitat within Ever-Wet Tropical Forest zones of 50% compared to original extent and an estimated future loss of 20% by 2050 [30]. The dataset included is the area of forest (>75%) remaining in the overlap between current and future ever wet zones. There is some uncertainty associated with the MODIS data and these areas have therefore been classified as potential Critical Habitat. | **Potential** |
|  | Tropical dry forests | Global distribution of Tropical dry forest [31] | ⚫ | 🞆 | Tropical dry forests are considered among the most threatened of the world’s forested ecosystems [57,58]; past deforestation rates range from 2-18% over a 20 year period, and close to 40% of forested areas are considered at severe risk from climate change [31]. As an example, the dry forests of some Pacific islands are thought to have been reduced to less than 10% of their original extent [59] and two thirds of tropical dry forest in the Americas has already been converted [58]. Therefore tropical dry forest in many regions of the world would likely approach the RLE threshold for threatened ecosystem based on past and future rates of loss. Due to regional variation this ecosystem type has been categorised as potential Critical Habitat. | **Potential** |
| Areas determined to be irreplaceable or of high priority/significance based on systematic conservation planning techniques carried out at the landscape and/or regional scale by governmental bodies, recognized academic institutions and/or other relevant qualified organizations (including internationally-recognized Non-Governmental Organizations NGOs) or that are recognized as such in existing regional or national plans, such as the NBSAP | - | - | - | - | - | - |
| **Criterion 5: Areas associated with key evolutionary processes** | | | | | | |
| The physical features of a landscape that might be associated with particular evolutionary processes | - | - | - | - | - | - |
| Subpopulations of species that are phylogenetically or morphogenetically distinct and may be of special conservation concern given their distinct evolutionary history. The latter includes Evolutionarily Significant Units (ESUs) and Evolutionarily Distinct and Globally Endangered (EDGE) species | - | - | - | - | - | - |
| **Scenario A: Other recognized high biodiversity values that might also support a Critical Habitat designation (examples)** | | | | | | |
| Areas required for the reintroduction of CR and EN species and refuge sites for these species (e.g. habitat used during periods of stress such as flood, drought or fire) | - | - | - | - | - | - |
| Ecosystems of known special significance to EN or CR species for climate adaptation purpose | - | - | - | - | - | - |
| Concentrations of Vulnerable (VU) species in cases where there is uncertainty regarding the listing, and the actual status of the species may be EN or CR | - | - | - | - | - | - |
| Areas of primary/old-growth/pristine forests and/or other areas with especially high levels of species diversity | - | - | - | - | - | - |
| Landscape and ecological processes, such as water catchments, areas critical to erosion control, disturbance regimes (e.g., fire, flood), that are required for maintaining Critical Habitat | - | - | - | - | - | - |
| Habitat necessary for the survival of keystone species | - | - | - | - | - | - |
| Areas of high scientific value such as those containing concentrations of species new and/or little known to science | - | - | - | - | - | - |
| **Scenario B: Internationally and/or nationally recognized areas of high biodiversity value that in general will likely qualify as Critical Habitat (examples)** | | | | | | |
| Areas that meet the criteria of the IUCN’s Protected Area Management Categories Ia, Ib and II | Protected areas under IUCN management categories Ia, Ib and II | World Database on Protected Areas [22] | ⚫ | ⚫ | Direct mention in GN6. | **Likely**. |
| UNESCO natural World Heritage sites that are recognized for their Global Outstanding Value | UNESCO World Heritage sites | World Database on Protected Areas [22] | ⚫ | ⚫ | Direct mention in GN6. | **Likely** |
| The majority of Key Biodiversity Areas (KBAs), which encompass inter alia Ramsar Sites, Important Bird Areas (IBA), Important Plant Areas (IPA) and AZE (GN115: Within the list of recognized areas of high biodiversity value provided in paragraph GN57, legally protected areas with an IUCN Management Category of Ia, Ib and II, UNESCO Natural World Heritage Sites and Ramsar sites will be treated as critical habitat. | Ramsar sites | World Database on Protected Areas [22] | ⚫ | ⚫ | Direct mention in GN6. | **Likely** |
| Areas determined to be irreplaceable or of high priority/significance based on systematic conservation planning techniques carried out at the landscape and/or regional scale by governmental bodies, recognized academic institutions and/or other relevant qualified organizations (including internationally-recognized NGOs) | Irreplaceable Protected Areas | Protected Areas as identified in [21], data drawn from the World Database on Protected Areas [22] | ⚫ | ⚫ | 137 PAs were identified by combining the 100 highest-ranking sites in terms of overall irreplaceability with the 100 most irreplaceable areas for threatened species. 88 of the combined set were previously identified as holding ≥95% of the global population of at least one highly threatened species. Study conducted by individuals from multiple conservation organisations, including IUCN, UNEP-WCMC, and Conservation International. | **Likely** |
| Areas identified by the client as High Conservation Value (HCV) using internationally recognized standards, where criteria used to designate such areas is consistent with the high biodiversity values listed in the five Critical Habitat criteria | - | - | - | - | - | - |

**References**

*Numbering of references is aligned with and continuous to the numbering used in the article. References [22] to [43] are also cited within the article.*

22. IUCN and UNEP-WCMC. The World Database on Protected Areas (WDPA), February 2017 version. Available from: [www.protectedplanet.net](http://www.protectedplanet.net)

23. IUCN. The IUCN Red List of Threatened Species. Version 2016-1. Available from: [www.iucnredlist.org](http://www.iucnredlist.org)

24. BirdLife International. World Database of Key Biodiversity Areas. Developed by the KBA Partnership. 2016. Available from: [www.keybiodiversityareas.org](http://www.keybiodiversityareas.org)

28. Giri C, Ochieng E, Tieszen LL, Zhu Z, Singh A, Loveland T, et al. Status and distribution of mangrove forests of the world using earth observation satellite data (version 1.3, updated by UNEP-WCMC). Glob Ecol Biogeogr. 20:154–9.

29. UNEP-WCMC, Conservation International, The Nature Conservancy. Global distribution of saltmarsh (ver. 4.0). Cambridge (UK): UNEP World Conservation Monitoring Centre; Available from: <http://data.unep-wcmc.org/datasets/43>

30. Underwood EC, Olson D, Hollander AD, Quinn JF. Ever-wet tropical forests as biodiversity refuges. Nat Clim Chang. 2014 Aug 27;4(9):740–1.

31. Miles L, Newton AC, DeFries RS. A global overview of the conservation status of tropical dry forests. Journal of Biogeography. 2006; Available from: <http://onlinelibrary.wiley.com/doi/10.1111/j.1365-2699.2005.01424.x/full>

33. Aldrich et al. A Global Directory of Tropical Montane Cloud Forests. 1997.

35. UNEP-WCMC. Global distribution of sea turtle nesting sites (version 1.1). Cambridge (UK): UNEP World Conservation Monitoring Centre; 1999 [cited 2016 Jun 1]. Available from: <http://data.unep-wcmc.org/datasets/22>

36. Walston J, Robinson JG, Bennett EL, Breitenmoser U, da Fonseca GAB, Goodrich J, et al. Bringing the tiger back from the brink-the six percent solution. PLoS Biol . 2010 Sep 14;8(9). Available from: <http://dx.doi.org/10.1371/journal.pbio.1000485>

43. Safi K, Armour-Marshall K, Baillie JEM, Isaac NJB. Global patterns of evolutionary distinct and globally endangered amphibians and mammals. PLoS One. 2013 May 15;8(5):e63582

49. IUCN. 2016. Guidelines for the application of IUCN Red List of Ecosystems Categories and Criteria Bland, L.M., Keith, D.A., Miller, R.M., Murray, N.J. & Rodríguez, J.P. (eds.) Version 1.0. Gland, Switzerland: IUCN. ix + 99pp.

50. Duarte CM, Dennison WC, Orth RJW, and Carruthers TJB. (2008). The charisma of coastal ecosystems: addressing the imbalance. Estuaries and Coasts 31: 233–38

51. Nellemann C, Corcoran E, Duarte CM, Valdes L, De Young C, Fonseca L, Grimsditch G (Eds.) (2009). Blue carbon. A rapid response assessment. Nairobi (Kenya): United Nations Environment Programme, and Arendal (Norway): GRID-Arendal. 80 pp.

52. Crooks S, Herr D., Tamelander T, Laffoley D, Vandever J (2011). Mitigating climate change through restoration and management of coastal wetlands and near-shore marine ecosystems. Challenges and opportunities. Environment Department Paper 121. Washington (DC): World Bank. 69 pp.

53. Spalding et al. 2010 (Mangroves) - Spalding MD, Meliane I, Milam A, Fitzgerald C, Hale LZ (2013). Protecting marine spaces: global targets and changing approaches. Ocean Yearbook 27: 213-248

54. FAO (2003). Status and trends in mangrove area extent world- wide. By Wilkie ML and Fortuna S. Forest Resources Assessment Working Paper No. 63. Rome (Italy): Forest Resources Division of the Food and Agricultural Organization of the United Nations

55. McClenachan L, Jackson JBC, Newman MJH (2006) Conservation implications of historic sea turtle nesting beach loss. Front Ecol Environ 4:290–296

56. Mulligan M. 2010. Modeling the tropics-wide extent and distribution of cloud forest and cloud forest loss, with implications for conservation priority.In: Bruijnzeel LA, Scatena FN, Hamilton LS, eds. Tropical montane cloud forests. Science for conservation and management. Cambridge: Cambridge University Press, 14–39.

57. Olson, D.M., Dinerstein, E., Abell, R., Allnutt, T., Carpenter, C., McClenachan, L., D’Amico, J., Hurley, P, Kassem, K., Strand, H., Taye, M. & Thieme, M. (2000) The global 200: a representation approach to conserving the Earth’s distinctive ecoregions. Conservation Science Program, World Wildlife Fund-US, Washington.

58. Blackie R, Baldauf C, Gautier D, Gumbo D, Kassa H, Parthasarathy N, Paumgarten F, Sola P, Pulla S, Waeber P and Sunderland T. 2014. Tropical dry forests: The state of global knowledge and recommendations for future research.Discussion Paper. Bogor, Indonesia: CIFOR.

59. Gillespie TW, Lipkin B, Sullivan L, Benowitz DR, Pau S, Keppel G. 2012. The rarest and least protected forests in biodiversity hotspots. Biodivers Conser 21:3597-3611. DOI 10.1007/s10531-012-0384-1.

71. Olson, D. M., E. Dinerstein, E. Wikramanayake, N. Burgess, G. Powell, E. C. Underwood, J. D'Amico, I. Itoua, H. Strand, J. Morrison, C. Loucks, T. Allnutt, T. H. Ricketts, Y. Kura, W. Wettengel, and K. Kassem. 2001. Terrestrial ecoregions of the world: a new map of life on earth. Bioscience. BioScience 51:933-938.

74. Ellis EC, Ramankutty N (2008) Putting people in the map: Anthropogenic biomes of the world. Version 1. Front Ecol Environ. 6(8): 439-447, doi: 10.1890/070062

75. US Fish and Wildlife Service. 2015. Critical Habitat for Threatened & Endangered Species [USFWS].

76. UNEP-WCMC (2010). Global distribution of islands. Global Island Database (version 1). UNEP World Conservation Monitoring Centre. Based on version 1 of Wessel and Smith (1996) [78]. Full technical documentation is in Depraetere (2007) [79]. Cambridge (UK): UNEP World Conservation Monitoring Centre.

77. Mallet et al 2016. ISEAL Report: How sustainability standards can contribute to landscape approaches and zero deforestation commitments.

78. Wessel P, Smith WHF (1996). A global, self-consistent, hierarchical, high-resolution shoreline database. Journal of Geophysical Research 101: 8741-8743

79. Depraetere C (2007). IBPoW Database. A technical note on a global dataset of islands. Global Islands Network. 58 pp.

1. [↑](#footnote-ref-1)
2. CR, EN and VU refers to the IUCN Red List categories of Critically Endangered (CR), Endangered (EN) and Vulnerable (VU) species. [↑](#footnote-ref-2)
3. GN6 does not provide detailed numeric thresholds to identify highly threatened and unique ecosystems, but points to the IUCN Red List of Ecosystems (RLE) as a suitable standard in development (paragraph GN62). The proposed RLE provides quantitative guidelines to assess the threatened status of ecosystems, in Bland et al. (2016). For the purpose of justifying the inclusion of a particular ecosystem type in the screening layer we assessed whether the past or future rates of loss were estimated to be greater than the proposed threshold for a Vulnerable ecosystem of ≥30% loss in present and future, or ≥50% loss over historic time periods (Criteria A). The other RLE criteria for restricted distribution, environmental degradation, disruption of biotic processes, and quantitative analysis that estimates the probability of ecosystem collapse, were not considered possible to assess at a global scale at present. The ecosystems considered for inclusion were selected based on known available global data sources. [↑](#footnote-ref-3)
