# Supplementary material for: Global screening for Critical Habitat in the terrestrial realm
Source: PLoS One. 2018 Mar 22;13(3):e0193102. doi: 10.1371/journal.pone.0193102 (PMC5863962; doi:10.1371/journal.pone.0193102)
Supplement: S2 Table — Includes information on which element of the PS6 Critical Habitat they align with, the dataset investigated and the reason it was excluded from the global screening layer. (DOCX) [file pone.0193102.s002.docx]

**S2 Table. Further datasets considered and justification for exclusion**. Includes information on which element of the PS6 Critical Habitat they align with, the dataset investigated and the reason it was excluded from the global screening layer.

| **Description** | **Relevant**  **biodiversity feature** | **Dataset(s) reference** | **Justification for exclusion** |
| --- | --- | --- | --- |
| **Criterion 1: Habitats of significant importance to Critically Endangered (CE) and/or Endangered (EN) species** | | | |
| **C1, Tier 1** | | | |
| a) Habitat required to sustain ≥ 10% of the global population of an IUCN Red-listed CR or EN species where there are known, regular occurrences of the species and where that habitat could be considered a discrete management unit for that species | IPAs designated under criterion A1 | Important Plant Areas [60] | Spatial data are not global and designation is undertaken by individual national partners who hold data and control access. There is potential to include these data in future iterations of the screening layer. |
| b) Habitat with known, regular occurrences of CR or EN species where that habitat is one of 10 or fewer discrete management sites globally for that species | - | - | - |
| **C1, Tier 2** | | | |
| c) Habitat that supports the regular occurrence of a single individual of an IUCN Red-listed CR species and/or habitat containing regionally-important concentrations of an IUCN Red-listed EN species where that habitat could be considered a discrete management unit for that species | IUCN range maps for CR and EN species | Spatial data for the Red List of Threatened Species [23, 48] | Spatial data too coarse and not currently refined based on habitat suitability. As Extent of Suitable Habitat maps become available there is potential to include these data in future iterations of the screening layer. |
|  | IPAs designated under criterion A1 | Important Plant Areas [60] | Spatial data are not global and designation is undertaken by individual national partners who hold data and control access. There is potential to include these data in future iterations of the screening layer. |
| d) Habitat of significant importance to CR or EN species that are wide-ranging and/or whose population distribution is not well understood and where the loss of such a habitat could potentially impact the long-term survivability of the species | *-* | *-* | *-* |
| e) As appropriate, habitat containing nationally/regionally-important concentrations of an EN, CR or equivalent national/regional listing | *-* | *-* | *-* |
| **Criterion 2: Habitats of significant importance to endemic and/or restricted-range species** | | | |
| **C2, Tier 1** | | | |
| a) Habitat known to sustain ≥ 95% of the global population of an endemic or restricted-range species where that habitat could be considered a discrete management unit for that species (e.g., a single-site endemic) | - | - | - |
| **C2, Tier 2** | | | |
| b) Habitat known to sustain ≥ 1% but < 95% of the global population of an endemic or restricted-range species where that habitat could be considered a discrete management unit for that species, where adequate data are available and/or based on expert judgment | IUCN range maps for species which occupy ≤ 50,000km2 | Spatial data for the Red List of Threatened Species [23] | Spatial data too coarse and not currently refined based on habitat suitability. As Extent of Suitable Habitat maps become available there is potential to include these data in future iterations of the screening layer. |
|  | Centres of Plant Diversity which occupy ≤ 50,000km2 | Centres of Planet Diversity. Version 1.0 [61] | These data are too course to indicate presence of this feature at a 1km^2^ resolution. |
|  | IPAs designated under criterion A4 | Important Plant Areas [60] | Spatial data are not global and designation is undertaken by individual national partners who hold data and control access. There is potential to include these data in future iterations of the screening layer. |
|  | Endemic Birds Areas | Endemic Bird Areas [80] | Endemic Bird Areas are a regional-scale designation. Due to the large extent of such designations, the biodiversity values are unlikely to be represented continuously throughout the designated area. |
| **Criterion 3: Habitats supporting globally significant concentrations of migratory species and/or congregatory species** | | | |
| **C3, Tier 1** | | | |
| a) Habitat known to sustain, on a cyclical or otherwise regular basis, ≥ 95% of the global population of a migratory or congregatory species at any point of the species’ life-cycle where that habitat could be considered a discrete management unit for that species | *-* | - | *-* |
| **C3, Tier 2** | | | |
| b) Habitat known to sustain, on a cyclical or otherwise regular basis, ≥ 1% but < 95% of the global population of a migratory or congregatory species at any point of the species’ life-cycle and where that habitat could be considered a discrete management unit for that species, where adequate data are available and/or based on expert judgment | Migratory Species | Global Register of Migratory Species, [62] | These data are too coarse to indicate presence of this feature at a 1km^2^ resolution. |
|  | Polar congregatory species | Environmental & Research Assessment (ERA) dataset of congregatory species ranges | A GIS database of Antarctic birds, including ranges of congregatory species, has been compiled by ERA but this is not publicly available |
| c) For birds, habitat that meets BirdLife International’s criterion A4 for congregations and/or Ramsar Criteria 5 or 6 for Identifying Wetlands of International Importance | - | - | - |
| d) For species with large but clumped distributions, a provisional threshold is set at ≥ 5% of the global population for both terrestrial and marine species | - | - | **-** |
| e) Source sites that contribute ≥ 1% of the global population of recruits | - | - | **-** |
| **Criterion 4: Highly threatened and/or unique ecosystems** | | | |
| Highly threatened or unique ecosystems are those:   - that are at risk of significantly decreasing in area or quality; - with a small spatial extent; and/or - containing unique assemblages of species including assemblages or concentrations of biome-restricted species | IUCN Red List of Ecosystems | Spatial data for case studies in [27] | Information currently only available on 20 case studies. There is potential to include these data as they become further available in future iterations of the screening layer. |
|  | Crisis ecoregions | [81] | These data are too coarse to indicate presence of this feature at a 1km^2^ resolution |
|  | IPAs designated under criterion C | Important Plant Areas [60] | Spatial data are not global and designation is undertaken by individual national partners who hold data and control access. There is potential to include these data in future iterations of the screening layer. |
|  | Karst landscapes | Karst Regions of the World [63]  *or*  World Map of Carbonate Rock Outcrops [64] | Data are available but very low in resolution. Not all Karst regions would be aligned with this criterion and these data would therefore need to be refined. |
|  | Mountain top ecosystems | Mountains of the world revisited [65] | Using elevation alone, available through the mountains of the world dataset, was not considered a valid approach as the threshold of high elevation would need to be determined regionally. Using global thresholds classifies large areas of high elevation land that would not be considered unique or vulnerable and omits important areas that would be determined as high elevation in that region. |
|  | Wetlands | Global Lakes and Wetlands Database [66] | Davidson (2014) [67] provides a global assessment of the published evidence for temporal and geographical trends in the extent of wetlands, and rates of change in wetland areas, based on a comprehensive literature review of 189 papers that resulted in 64 long term and 125 20 - early 21^st^ century records of change. The selected papers are published since 1982, and range in scale from local to global; coverage is patchy and limited, especially for Africa, the Neotropics and Oceania. Looking at wetlands as a generic set of ecosystems, Davidson (2014) provides quantitative evidence that at a global level they would qualify as at least Vulnerable under RLE criteria A: “the reported long-term loss of natural wetlands averages between 54–57% but loss may have been as high as 87% since 1700 AD. There has been a much (3.7 times) faster rate of wetland loss during the 20th and early 21st centuries, with a loss of 64–71% of wetlands since 1900 AD.” Wetlands are however comprised of a number of types with varying threat status and therefore were assessed individually for the wetland types for which global data exist, based on the data provided in Davidson (2014). |
|  | Peatlands, bogs and mires | Global Lakes and Wetlands Database [67] | A total of 23 records of change in peatlands, bogs and mires were reviewed by Davidson (2014) [67] of which 15 are focused on Europe. One publication provided a global estimate of change in peatland coverage of -0.95% change from 1990-2008 [68], with the highest regional estimates being shown for European peatlands, bogs, marshes and mires. Long-term assessments (<1900 AD) average at -71.8% total loss for localities assessed in Europe, and -43% for SE Asia. More recent assessments (>1947) average at -18.9% and have been completed on all continents. Variance across localities is large, the % change ranging from 78% in the Central Kalimantan, to values of <0.1%.  Based on the proposed RLE criteria by Keith et al. (2013) [27] the reported rates of historic and present loss of these ecosystems would trigger a threatened status in many cases, particularly within Europe but there is insufficient evidence for the inclusion of a global dataset within the layer. |
|  | Freshwater swamp forest and regularly flooded freshwater forest | Global Lakes and Wetlands Database [66] | Data are available in Davidson (2014) [67] for ten areas (national and site) of freshwater marsh and floodplain around the world from Europe, Canada, Kenya and China. For those with historic trends dating back to pre 1900s (n=6) there was an average of 64.5% total loss recorded and 0.67% loss per year, and for those where the loss has been recorded since 1900 (n=4) there was an average of 1.49% loss per year. Based on the proposed RLE criteria by Bland et al. (2016) [49] the reported rates of historic and present loss of these ecosystems for which data are available would trigger a threatened status. However there are insufficient data points to justify inclusion of the global data layer. |
|  | Regularly flooded freshwater forest | Global forest map [69] | Data are available in Davidson (2014) [67] for six areas of swamp and flooded forest around the world from Europe, North America, Israel and China. For those with historic trends dating back to pre 1900s (n=2) there was an average total loss of 45.6% and for those where loss has been recorded since the 1900s (n=4) there was an average of 0.5% loss per year. Based on the proposed RLE criteria by Bland et al. (2016) [49] the reported rates of historic and present loss of these ecosystems for which data are available would trigger a threatened status. However there are insufficient data points to justify inclusion of the global data layer. |
|  | Mediterranean type ecosystems | Remaining natural areas in Mediterranean type ecoregions , developed by TNC [82] | Mediterranean type ecosystems, located between 30-40 degrees north and south of the equator, are in five locations around the world (California, Chile, South Africa, Southwestern Australia and the Mediterranean Basin). They cover <5% of the Earth’s surface but contain almost 50,000 known vascular plant species, almost 20% of the world total [70]. These Mediterranean type ecosystems, defined by the 39 Mediterranean ecoregions [71], The historic rates of loss of this ecosystem type range from 17-37 % for each of the regions [81] and therefore many regions would class as threatened based on the RLE historic rate of loss threshold [49]. These data were however excluded due to the spatial resolution of the dataset being too coarse. |
| Areas determined to be irreplaceable or of high priority/significance based on systematic conservation planning techniques carried out at the landscape and/or regional scale by governmental bodies, recognized academic institutions and/or other relevant qualified organizations (including internationally-recognized Non-Governmental Organizations NGOs) or that are recognized as such in existing regional or national plans, such as the NBSAP | Intact Forest Landscapes | [47] | These data cover large expanses of land that would include many areas of low significance and irreplaceability and they are therefore not considered suitably aligned with the definition. |
|  | Biodiversity Hotspots | [83] | These data are too coarse to indicate presence of this feature at a 1km^2^ resolution. |
| **Criterion 5: Areas associated with key evolutionary processes** | | | |
| The physical features of a landscape that might be associated with particular evolutionary processes | Islands | Global distribution of islands (WCMC) | No accepted thresholds for the definition of "isolated" for islands. |
|  | Lakes | Global Lakes and Wetlands Database [66] | While many lakes would be aligned with this definition, they are not suitably aligned at the global level. |
|  | Mountaintops | HydroSHEDS [72] | These data are insufficiently refined for this purpose. There is a universal threshold to link mountain height and slope to increased isolation which could be investigated for future iterations of the screening layer. |
|  | Areas of importance to climate change adaptation | [73] | Maps of species vulnerability to climate change are available but as these data are based on the IUCN range maps they are insufficiently refined to indicate presence of these species at a 1km^2^ resolution. These also do not indicate areas important for adaptation of species to climate change, only their vulnerability in response. |
| Subpopulations of species that are phylogenetically or morphogenetically distinct and may be of special conservation concern given their distinct evolutionary history. The latter includes Evolutionarily Significant Units (ESUs) and Evolutionarily Distinct and Globally Endangered (EDGE) species | High ED/EDGE species | Zoological Society of London (ZSL) dataset for mammals, amphibians and birds combined with  Spatial data for the Red List of Threatened Species [23] | Data for Evolutionary Distinct (ED) species considered to be most relevant. ED species ranges are too coarse in resolution for inclusion here. ZSL currently creating ED zones that control for species richness to indicate areas of high evolutionary distinctiveness. Potential to include in future iterations of the screening layer. |
| **Scenario A: Other recognized high biodiversity values that might also support a Critical Habitat designation (examples)** | | | |
| Areas required for the reintroduction of CR and EN species and refuge sites for these species (e.g. habitat used during periods of stress such as flood, drought or fire) | *-* | - | - |
| Ecosystems of known special significance to EN or CR species for climate adaptation purpose | - | - | - |
| Concentrations of Vulnerable (VU) species in cases where there is uncertainty regarding the listing, and the actual status of the species may be EN or CR | *-* | - | - |
| Areas of primary/old-growth/pristine forests and/or other areas with especially high levels of species diversity | Intact forests | Intact Forest Landscapes [47] | These data cover large expanses of land that would include many areas of low significance and they are therefore not considered suitably aligned with the definition. |
| Landscape and ecological processes, such as water catchments, areas critical to erosion control, disturbance regimes (e.g., fire, flood), that are required for maintaining Critical Habitat | *-* | - | - |
| Habitat necessary for the survival of keystone species | *-* | - | - |
| Areas of high scientific value such as those containing concentrations of species new and/or little known to science | *-* | - | - |
| **Scenario B: Internationally and/or nationally recognized areas of high biodiversity value that in general will likely qualify as Critical Habitat (examples)** | | | |
| Areas that meet the criteria of the IUCN’s Protected Area Management Categories Ia, Ib and II, although areas that meet criteria for Management Categories III-VI may also qualify depending on the biodiversity values inherent to those sites | - | - | Protected areas that meet IUCN category III-VI and those that are unreported were excluded due to the lack of knowledge on the underlying criteria on which they were designated |
| UNESCO natural World Heritage sites that are recognized for their Global Outstanding Value | - | - | - |
| The majority of Key Biodiversity Areas (KBAs), which encompass inter alia Ramsar Sites, Important Bird Areas (IBA), Important Plant Areas (IPA) and AZE | - | - | - |
| Areas determined to be irreplaceable or of high priority/significance based on systematic conservation planning techniques carried out at the landscape and/or regional scale by governmental bodies, recognized academic institutions and/or other relevant qualified organizations (including internationally-recognized NGOs) |  |  |  |
| Areas identified by the client as High Conservation Value (HCV) using internationally recognized standards, where criteria used to designate such areas is consistent with the high biodiversity values listed in the five Critical Habitat criteria | Some certification schemes require HCV areas to be mapped |  | Data largely unavailable and a global synthesis has not been carried out. |

**References**

*Numbering of references is aligned with and continuous to the numbering used in the article. References [22] to [47] are cited within the article.*

23. IUCN. The IUCN Red List of Threatened Species. Version 2016-1. Available from: [www.iucnredlist.org](http://www.iucnredlist.org)

27. Keith DA, Rodríguez JP, Rodríguez-Clark KM, Nicholson E, Aapala K, Alonso A, et al. Scientific foundations for an IUCN Red List of ecosystems. PLoS One. 2013 May 8;8(5):e62111.

43. Plantlife International (2004) Identifying and Protecting the World’s Most Important Plant Areas: A Guide to Implementing Target 5 of the Global Strategy for Plant Conservation. Salisbury, UK: Plantlife International.

47. Potapov P, Yaroshenko A, Turubanova S, Dubinin M, Laestadius L, Thies C, et al. Mapping the world’s intact forest landscapes by remote sensing. Ecol Soc . 2008;13(2). Available from: http://www.ecologyandsociety.org/vol13/iss2/art51/main.html

48. BirdLife International and NatureServe (2015) *Bird species distribution maps of the world*. Version 5.0. BirdLife International, Cambridge, UK and NatureServe, Arlington, USA.

49. IUCN. 2016. Guidelines for the application of IUCN Red List of Ecosystems Categories and Criteria Bland, L.M., Keith, D.A., Miller, R.M., Murray, N.J. & Rodríguez, J.P. (eds.) Version 1.0. Gland, Switzerland: IUCN. ix + 99pp.

61. UNEP-WCMC 2013. Centres of Plant Diversity. Version 1.0 (digital reproduction of Centres of Plant Diversity, eds S.D. Davis, V.H. Heywood & A.C. Hamilton, WWF and IUCN, Gland, Switzerland, 1994-7)

62. GROMS, Riede 2004) - Riede, Klaus (ed) (2004): Global Register of Migratory Species - from Global to Regional Scales. Final Report of the R&D-Projekt 808 05 081. Supported by the Federal Agency for Nature Conservation, with funds from the German Federal Ministry for the Environment, Nature Conservation and Nuclear Safety. In cooperation with the UNEP Secretariat (Bonn) for the Convention on the Conservation of Migratory Species of Wild Animals and the Alexander Koenig Research Institute and Museum of Zoology - Leibnitz Institute for Research in Terrestrial Biodiversity.

63. Karst Regions of the World, Hollingsworth 2009 0 Hollingsworth, E.J. (2009) Karst Regions of the World (KROW) – Populating Global Karst Datasets and Generating Maps to Advance the Understanding of Karst Occurrence and Protection of Karst Species and Habitats Worldwide (Master of Science in Geology). University of Arkansas, Fayetteville, AR.

64. Williams PW, 2007, World Map of Carbonate Rock outcrops (v 3.0) University of Auckland, New Zealand. Website: <http://web.env.auckland.ac.nz/our_research/karst/> ; <http://digital.lib.usf.edu/SFS0055342/00001>

65. FAO, UNEP-WCMC, University of Bern (2014) Mountains of the World Revisited.

66. Lehner, B. and Döll, P. (2004): Development and validation of a global database of lakes, reservoirs and wetlands. Journal of Hydrology 296/1-4: 1-22.

67. Davidson (2014) How much wetland has the world lost? Long-term and recent trends in global wetland area. Marine and Freshwater Research 65(10) 934-941.<https://doi.org/10.1071/MF14173>

68. Joosten, H. (2009). The Global Peatland CO2 Picture. Peatland status and drainage related emissions in all countries of the world. (Wetlands International, Ede, The Netherlands.)

69. Schmitt CB, Burgess ND, Coad L, Belokurov A, Besancon C, Boisrobert L, Campbell A, Fish L, Gliddon D, Humphries K, Kapos V, Loucks C, Lysenko I, Miles L, Mills C, Minnemeyer S, Pistorius T, Ravilious C, Steininger M, Winkel G (2009) Global analysis of the protection status of the world’s forest. Biological Conservation. Volume 142, Issue 10, pages 2122-2130.

70. Cowling, R. M., P. W. Rundel, B. B. Lamont, M. K. Arroyo, and M. Arianoutsou. 1996. Plant diversity in mediterranean climate regions. Trends in Ecology and Evolution 11:362-366.

72. Lehner, B., Verdin, K., Jarvis, A. (2008): HydroSHEDS. New global hydrography derived from spaceborne elevation data. Eos, Transactions, AGU, 89(10): 93-94. Credits: WWF. Data download: http://hydrosheds.cr.usgs.gov.

73. Foden, W.B. et al. (2013) Identifying the World's Most Climate Change Vulnerable Species: A Systematic Trait-Based Assessment of all Birds, Amphibians and Corals. PLoS ONE, 8, e65427.

80. BirdLife International, 1998. Endemic Bird Areas - digital boundaries. Downloaded under licence from the Integrated Biodiversity Assessment Tool. http://www.ibatforbusiness.org

81. Hoekstra, J. M., Boucher, T. M., Ricketts, T. H. & Roberts, C. 2005 Confronting a biome crisis: global disparities of habitat loss and protection. Ecol. Lett. 8, 23–29.

82. Underwood, E. C., J. H. Viers, K. R. Klausmeyer, R. L. Cox, and M. R. Shaw. 2009. Threats and biodiversity in the Mediterranean Biome. Diversity and Distributions 15:188-197.

83. Conservation International, 2011. Biodiversity Hotspots - digital boundaries. Downloaded under licence from the Integrated Biodiversity Assessment Tool. http://www.ibatforbusiness.org
