# Supplementary material for: Global screening for Critical Habitat in the terrestrial realm
Source: PLoS One. 2018 Mar 22;13(3):e0193102. doi: 10.1371/journal.pone.0193102 (PMC5863962; doi:10.1371/journal.pone.0193102)
Supplement: S3 Table — (DOCX) [file pone.0193102.s003.docx]

**S3 Table: The proposed alignment between the KBA criteria in the new Global Standard for the identification of KBAs (IUCN 2016) and IFC Critical Habitat (CH) criteria.**

| **KBA criteria and thresholds** | **Proposed CH classification** | **PS6**  **criterion** |
| --- | --- | --- |
| **A. Threatened biodiversity element at site** | | |
| **A1. Threatened species** | | |
| (a) CR or EN species ≥0.5% & ≥5 reproductive units (RU) | Likely | 1a |
| (b) VU species ≥1% & ≥10 RU | None |  |
| (c) CR or EN species threatened only due to population size reduction in the past or present ≥0.1% & ≥5 RU | Likely | 1b |
| (d) VU species threatened only due to population size reduction in the past or present ≥0.2% & ≥10 RU | None |  |
| (e) CR or EN species - Entire global population size | Likely | 1a |
| **A2: Threatened ecosystem types** |  |  |
| (a) CR or EN ecosystem type ≥5% | Likely | 4 |
| (b) VU ecosystem type ≥10% | None | 4 |
| **B. Geographically restricted biodiversity element at site** | | |
| B1: Individual geographically restricted species: any species ≥10% & ≥10 RU | Likely | 2b |
| B2: Co-occurring geographically restricted species: ≥2 species OR 0.02% of total number of species in taxonomic group, whichever is larger, ≥1% of each | Likely | 2b |
| **B3: Geographically restricted assemblages** | | |
| (a) ≥5 ecoregion-restricted species OR 10% of the species restricted to the ecoregion, whichever is larger; ≥0.5% of each | Potential | Sc B/4 |
| (b) ≥5 bioregion-restricted species OR 30% of the bioregion-restricted species known from the country, whichever is larger | Potential | Sc B/4 |
| (c) Part of the globally most important 5% of occupied habitat of each of ≥5 species within a taxonomic group | Potential | Sc B/4 |
| **B4: Geographically restricted ecosystem types** | | |
| Any ecosystem type ≥20% | Likely | 4 |
| **C. Ecological integrity** *Biodiversity element at site* | | |
| Wholly intact ecological communities ≤2 sites per ecoregion | Likely | Sc A |
| **D. Biological processes** | | |
| D1: Demographic aggregations | | |
| (a) Species aggregation during one or more key stages of its life cycle ≥1% | Likely | 3b |
| (b) Among the largest 10 aggregations known for the species | Potential | 3b |
| D2: Ecological refugia: Species aggregations during periods of past, current or future environmental stress ≥10% | Likely | 3b/3d |
| D3: Recruitment sources | | |
| Propagules, larvae or juveniles maintaining high proportion of global population size ≥10% | Likely | 3e |
| **E: Irreplaceability through quantitative analysis** | | |
| Site has high irreplaceability measured by quantitative spatial analysis *Irrepl. Score* ≥0.90 on 0–1 scale | Likely | Sc B |
