# Supplementary material for: Global screening for Critical Habitat in the terrestrial realm
Source: PLoS One. 2018 Mar 22;13(3):e0193102. doi: 10.1371/journal.pone.0193102 (PMC5863962; doi:10.1371/journal.pone.0193102)
Supplement: S4 Table — (DOCX) [file pone.0193102.s004.docx]

**S4 Table:** Relevance of regional-scale designations to Critical Habitat criteria

| Selection criterion | Relevant regional-scale designation |
| --- | --- |
| Criteria 1. CR and EN species |  |
| Criteria 2. Endemic and/or restricted-range species | Biodiversity Hotspots  Centres of Plant Diversity (CPD)  Endemic Bird Areas (EBA)  Global 200 Ecoregions |
| Criteria 3. Migratory and/or congregatory species |  |
| Criteria 4. Highly threatened and/or unique ecosystems | Biodiversity Hotspots  Centres of Plant Diversity (CPD)  Crisis Ecoregions  Global 200 Ecoregions |
| Criteria 5. Key evolutionary processes | Centres of Plant Diversity (CPD)  Global 200 Ecoregions |
