# Supplementary material for: Global screening for Critical Habitat in the terrestrial realm
Source: PLoS One. 2018 Mar 22;13(3):e0193102. doi: 10.1371/journal.pone.0193102 (PMC5863962; doi:10.1371/journal.pone.0193102)
Supplement: S1 Fig — A GIS dataset of the terrestrial Critical Habitat screening layer is available on request for research and conservation purposes from information@unep-wcmc.org. (DOCX) [file pone.0193102.s005.docx]

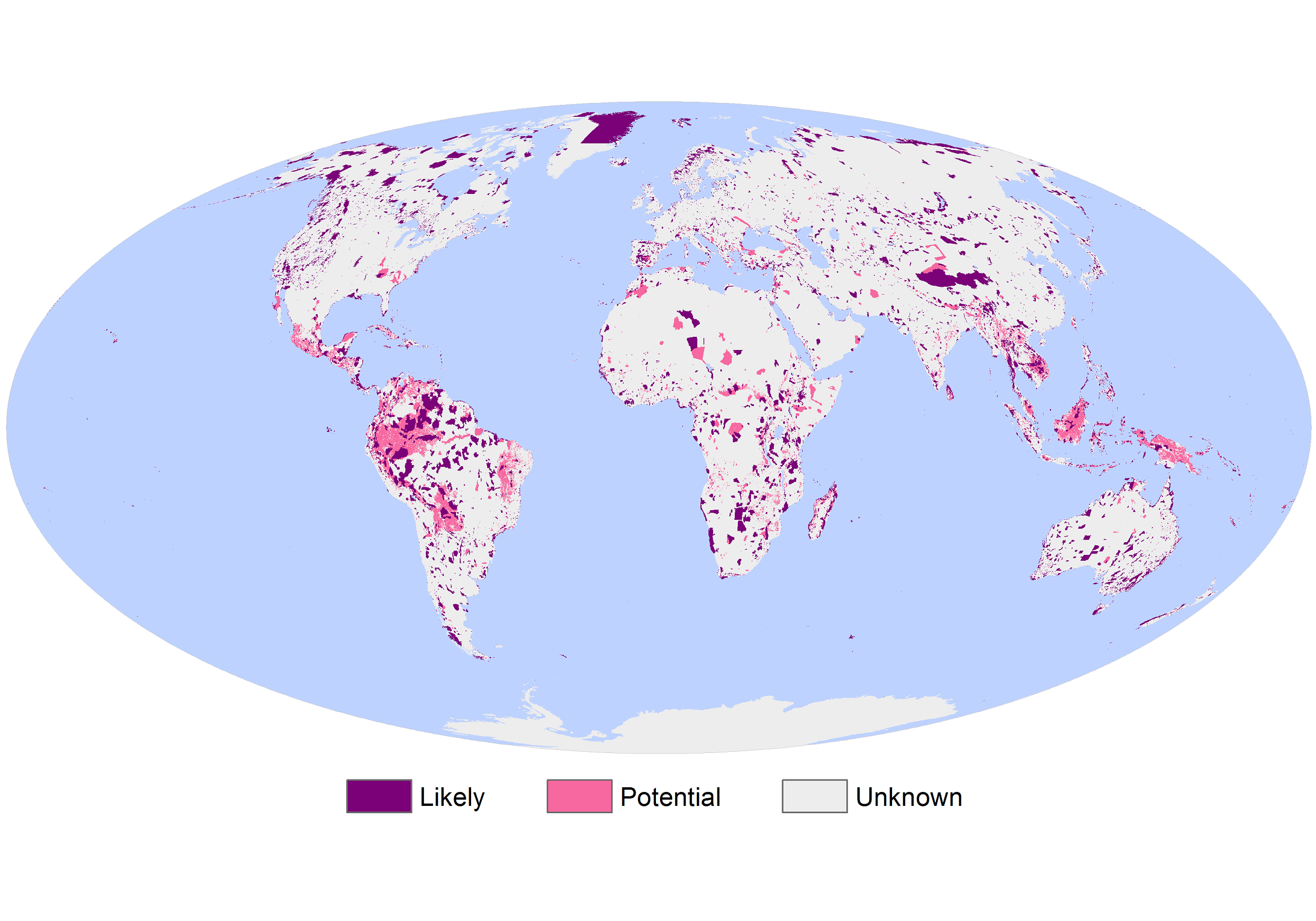
**S1 Fig. Global screening layer (1x1 km raster) for terrestrial Critical Habitat.** A GIS dataset of the terrestrial Critical Habitat screening layer is available on request for research and conservation purposes from [information@unep-wcmc.org](mailto:information@unep-wcmc.org).
